# Supplementary material for: Phylogenetic evidence reveals early Kra-Dai divergence and dispersal in the late Holocene
Source: Nat Commun. 2023 Oct 30;14:6924. doi: 10.1038/s41467-023-42761-x (PMC10616200; doi:10.1038/s41467-023-42761-x)
Supplement: Supplementary file 1 — Supplementary Information [file 41467_2023_42761_MOESM1_ESM.pdf]

Supplementary Information

Title: Phylogenetic evidence reveals early Kra-Dai divergence and dispersal in the late Holocene

Running title: Reconstructing Kra-Dai language prehistory

Authors:

Yuxin Tao[1†], Yuancheng Wei[2†], Jiaqi Ge[3†], Yan Pan[4†], Wenmin Wang[5], Qianqi Bi[6], Pengfei Sheng[7], Changzhong Fu[5], Wuyun Pan[8, 9], Li Jin[1], Hong-Xiang Zheng[10*], Menghan Zhang[8, 10, 11*]

Affiliation:

1 State Key Laboratory of Genetic Engineering, Center for Evolutionary Biology, Human Phenome Institute, Zhangjiang Fudan International Innovation Center, School of Life Science, Fudan University, Shanghai, 200438, China

2 School of Chinese Language and Literature, Guangxi Minzu University, Guangxi Zhuang Autonomous Region, China

3 Department of Chinese Language and Literature, Fudan University, Shanghai, China

4 Department of Cultural Heritage and Museology, Fudan University, Shanghai, China

5 College of Nationalities, Guangdong Polytechnic Normal University, Guangdong, China

6 College of Communication, East China University of Political Science and Law, Shanghai, China

7 Institute of Archaeological Science, Fudan University, Shanghai, China

8 Institute of Modern Languages and Linguistics, Fudan University, Shanghai, China

9 Institute for Humanities and Social Science Data, School of Data Science, Fudan University, Shanghai, China

10 Ministry of Education Key Laboratory of Contemporary Anthropology, Department of Anthropology and Human Genetics, School of Life Sciences, Fudan University, Shanghai, China

11 Research Institute of Intelligent Complex Systems, Fudan University, Shanghai China

† Y.T., Y.W., J.G., and Y.P. contributed equally to this work.

*To whom correspondence should be addressed. E-mail: Menghan Zhang (mhzhang@fudan.edu.cn), Hong-Xiang Zheng (zhenghongxiang@fudan.edu.cn)

1. Supplementary Methods

1.1. Preparation of sources for lexical cognate data of Kra-Dai languages

The linguistic data used in our study were compiled from a large-scale lexical database of more than 100 Kra-Dai languages (hereafter Kra-Dai database). The Kra-Dai database synthesized several data sources including Starostin's cognate database (https://starling.rinet.ru/cgi-bin/main.cgi) derived from the Kra-Dai etymological databases compiled by Ilya Peiros[1], several recent public research reports (e.g., *An introduction to the Kam-Tai languages (Chinese version)*[2]), and the first-hand language documents from the linguistic fieldworks conducted by authors (Supplementary Data 8). Our Kra-Dai database described the details for the lexicon of the 119 Kra-Dai languages at varying levels and maintained data expansion now. Then, 100 languages were screened out based on accuracy and coverage. Despite the heterogeneous data source in our Kra-Dai database, the lexical items covered the traditional items of basic vocabulary such as the Swadesh 100-word list. According to the Swadesh 100-word list, we integrated the lexical data into a big table in which each row was a language entry, meanwhile, each column was the International Phonetic Alphabet (IPA) transcription (Supplementary Data 1, sheet name "Lexical items") or lexical cognate trait (Supplementary Data 1, sheet name "Binary coded sets"). Our database would be a valuable reference for future linguistic studies across language families.

1.2. The summary of processing the lexical cognate identification

Following the historical linguistic definition[3], we adopted the terminology of "cognates" or "lexical cognates" in this study which referred to sets of words in different languages inherited in direct descent from an etymological ancestor in a common parent language (i.e., a proto-language). According to traditional linguistic views, there are five well-known branches in the Kra-Dai languages and five corresponding proto-languages at the branch level, respectively. In particular, the Proto-Tai language is considered the most recent common ancestor (MRCA) of Zhuang, Thai, Saek, and other languages in the Tai branch[4-8]. Proto-Kam-Sui is the MRCA of Kam, Sui, Mak, Then, and other languages in the Kam-Sui branch [1,9-15]. Note that Proto-Lakkia[16] including two child languages of Biao and Lakkia is involved in the Kam-Sui branch in our study. Proto-Hlai is the MRCA of Hlai, Ngan fon, Nadou, and other languages in the Hlai branch[17-19]. Proto-Ong-Be is the MRCA of contemporary Ong-Be languages[20]. And Proto-Kra is the MRCA of Gelao, Buyang, Lachi, and other languages in the Kra branch [21]. Proto-Kra-Dai language is defined as the MRCA of Proto-Tai, Proto-Kam-Sui, Proto-Ong-Be, Proto-Hlai, and Proto-Kra languages[1,2]. This presents the hierarchical structure of the Kra-Dai language family defined in our study. In line with the requirements of the comparative method of historical linguistics, the linguistic evidence in these previous studies provided not only identified lexical etymologies and shared morphology but also regular sound correspondences in each of the five branches. Such evidence further helped us to identify and label the lexical cognates across all Kra-Dai languages sampled in our study.

Accordingly, the regular sound correspondences of Kra-Dai languages involved four aspects: consonant, vowel, coda, and tone. Then, we used the traditional comparative methods[22] and internal

reconstruction[23] in historical linguistics to compare the lexical items (especially morphemes) to find out whether a corresponding group presents in given languages and identify the cognates of each item in the Swadesh 100-word list. We separated the identified lexical cognates for each meaning item into different lines where each line referred to a distinct cognate set. The IPA transcriptions of lexical items of Kra-Dai languages were listed in the corresponding lines of a cognate set when these languages shared the same cognate set (Supplementary Data 1. Linguistic Data, sheet name "Lexical items"). In this sheet, we left the cell blank if the lexical cognate was absent and labeled 28 cells with "provisional". Finally, we obtained a raw data table including 100 languages (columns) and 646 cognate sets (rows).

Then, the lexical cognate data of each language was transformed into a binary-coding table for further computational analysis (Supplementary Data 1. Linguistic Data, sheet name "Binary coded set"). Specifically, if a cognate existed in a language, we labeled this cognate set in this language as "1". If a cognate was not recorded in a language or was identified as a borrowing word, we considered that the cognate did not exist in this language and we would label a "0". When we could not confidentially identify a cognate set and considered it as a provisional one for a language, the codes of the cognate were labeled as "?". In the computational procedure, the "?" would be randomly taken as "0" and "1" with equal probabilities. To account for the fact that researchers tend not to collect data that does not vary, we used the ascertainment bias correction[24]. The first column for each aligned lexical item was an ascertainment column. There all languages had the entry "0" except the ones, where the data were missing, they have a "?"[25].

Here, we took the lexical item "eye" as an example. According to the regular sound correspondences of "eye", the consonants in Tai (/t/, /pr/, /p(j)/, /th/, /r(ɣ)/, etc.)[4], Ong-Be (/ɗ/)[20], Kam-Sui (/t/, /l̥/, /ⁿd/, /l/, etc.)[10,11], Hlai (/tsʰ/, /h/, /t/)[17], and Kra (/t/, /d/, /ð/, /ç/)[21] formed a diverse regular sound corresponding group. Meanwhile, the vowels and tones were neatly corresponding with each other, respectively. The vowels included /a/, /ɐ/, /ɔ/, their corresponding forms of vowel raising and diphthongization (e.g., /au/, /əu/, /iu/ in Kra branch[21]); whereas the tones were of tone 1 (A1), and the corresponding forms of tone divergence and merger (e.g., the change from 1 to 1' (A1'), 2 (A2), 6 (B2), 3 (C1) in Tai branch[4]). These correspondences indicated that they could share the same cognate set. However, only TnLianshan (Lianshan Zhuang dialect) was found to have the phonetic form /phat.8/. In contrast to the absence of plosive coda in other languages, /phat.8/ had a plosive coda and was of tone 8 (D2). These differences showed that /phat.8/ should be regarded as another cognate set. Therefore, after transforming to binary coded sets, there were two cognate sets of "*eye*". The first cognate set was "1" for all languages except "0" for TnLianshan, and the second was "1" for TnLianshan but "0" for other languages.

However, there are extra matters needed to be noticed in practice. The first one is to deal with compound words. In some Kra-Dai languages, polysyllabic forms are used to represent specific lexical items. As a result, a single word may be associated with two or more proto-roots. Thus, we must distinguish each morpheme of every lexical item as a single etymon and then we could apply linguistic comparative methods. For example, for each multi-morpheme compound word given a

language, firstly, we divided the word into distinctive morphemes and identify their etyma. Then, we found out which etymon presented most in all Kra-Dai languages. If this etymon was not in the Swadesh-100 core vocabulary list, it would substitute the original lexical item to be applied to comparative methods. For example, "*sun*" was a single morpheme in Indo-European languages, but a multi-morpheme compound word consisted of the etymon "*day*" in most Kra-Dai languages. As "*day*" was not in the Swadesh-100 core vocabulary list, we replaced "*sun*" with "*day*" in most languages. For cognate identification, for example, we first obtained the Proto-Tai root /*ŋwan/ for the etymon "day" from the previous linguistic studies. In PuBiao, we observed the multi-morpheme compound word /qa^{33} ɬaːŋ^{53}/ in our study of the lexical item of "*sun*". In this word, /ɬaːŋ^{53}/ could be the core morpheme related to "*sun*" because /qa^{33}/ was probably the nominalization of "*light*", which was inconsistent with the etymon "*sun*". Thus, we can linguistically compare the PuBiao's form /ɬaːŋ^{53}/ with the Proto-Tai root /*ŋwan/, and then conclude that they were not the same cognate set. The second matter is to connect the same morpheme shared by different lexical items with a single etymon. For example, to study the etymon "*night*" in Tai languages, we extracted the corresponding morphemes from the lexical items of "*nighttime*", "*evening*" and "*midnight*" and then identified two different proto-forms /*hɯn^{A2}/ and /*gam^{B2}/. Thus, we obtained two entries of cognates for /*hɯn^{A2}/ – *night*, and /*gam^{B2}/ – *night*.

The third one is to judge and exclude loanwords from the Kra-Dai database. The loanwords are recognized to be confounding factors for any type of linguistic comparative method to reconstruct the genealogical classification of a language tree and need to be identified and removed before applying the linguistic comparative method[26]. It is generally accepted that Kra-Dai languages experience a strong horizontal influence on Southern Chinese dialects. Even, in the Swadesh 100-word list, we could find that Chinese loanwords became replacing the indigenous words in some Kra-Dai languages[27-32]. In our lexical data of Kra-Dai languages, the loanwords were classified into two groups: one did not comply with regular sound correspondences; the others complied with regular sound correspondences in specific branches or clades of Kra-Dai languages. In particular, the former group should be excluded as it was clear to identify the source. For example, "moːt5" of lexical item "One" of TswNgheAn could be a loanword from Vietnamese; and "nak3" of lexical item "Black" of TswAiton could be a loanword from Burmese.

In contrast, the latter group was much more complicated. In our study, the approach of historical strata analysis[33] was performed to identify the lexical borrowings of Kra-Dai languages from Middle Chinese and modern Chinese dialects. For example, there existed numeral systems of Tai and Kam-Sui branches borrowed from that of Middle Chinese. Here, we took an example of the lexical item "One" in the Hlai, Ong-Be, Tai, and Kam-Sui branches. In particular, Hlai languages borrowed the phonetic form of "One" from the Hainan Min dialect; the Jizhao dialect of Ong-Be languages borrowed from the Wuchuan Yue dialect[30-32]; Tai languages borrowed from the Guangxi Pinghua dialect[27-29]; and Kam-Sui languages borrowed from either Gunagxi Pinghua Dialects or Southwestern Mandarin[29]. Although such systematic lexical borrowings between Kra-Dai languages and Chinese could be traced back to Middle Chinese, these were still excluded from our

study due to the heterogeneous sources. In addition, the case of "Heart" in the Kam-Sui branch, Central Tai and Northern Tai clades, and the case of "White" in the Central Tai clade were also regarded as loanwords from Chinese dialects. Notably, Zhang[27-29] pointed out that these loanwords were mainly derived from Guangxi Pinghua dialect in the late stage of Kra-Dai language formation. Despite the presence of systematical regular sound correspondences in several branches and clades, we still excluded these loanwords in practice. Notably, although some words such as "head" and "work" were *alleged* to be borrowed from Old Chinese, we should not exclude them because they could be assimilated following the intrinsic sound changes within the Kra-Dai languages and form regular sound correspondences. Therefore, these potential loanwords could be assimilated following the intrinsic sound changes within Kra-Dai languages. It is also acknowledged that the source of borrowings was not limited to Sinitic languages. Kra-Dai languages could borrow from other regionally dominant languages distributed in South China and MSEA. These languages include Austroasiatic languages (e.g., Khmer, Vietnamese), Tibetan-Burman languages (e.g., Burmese, Yi language), Hmong-Mien languages (e.g., Hmong), and even other Kra-Dai languages (e.g., Bouyei, Thai)[34]. However, the study on the influence of non-Sinitic languages on Kra-Dai languages was lacking and was only at the initial stage, so we did not exclude these obscure loanwords. All in all, we excluded the loanwords borrowed from Middle Chinese and Modern Chinese dialects but maintained the obscure loanwords borrowed from Old Chinese and other non-Sinitic languages.

1.3. The classification and labeling of Kra-Dai languages

We labeled our Kra-Dai language samples followed by Glottolog, Ethnologue, and the references listed in Supplementary Data 8. With the accumulation of language documents, the classifications of some Kra-Dai language samples required more detailed verification. For example, above Swadesh 100, the arguments for the classification of Tai Ya (Honghe in Xing[35]) and Tai la (Yuanjiang in Zhou and Luo[36]) were still matter in the view of historical linguistics. In our language samples, TswYuanjiang (also named Tai Ya) was classified as a Dai dialect and was part of the Southwestern Tai group. However, some linguistic materials inferred that Yuanjiang might share more phonological innovations with Central Tai rather than Southwestern Tai, and thus should be classified as Central Tai[6,37-39]. For example, the /kʰ/ in the word "*stream*" and "*laugh*" of Yuanjiang was consistent with that of Central Tai. The /tsʰ/ in the word "*shower (n.)*", "*six*" and "*ear*" of Yuanjiang were more similar to the /tɕʰ/ of Central Tai rather than the /h/ of Southwestern Tai. The change of /h-/ in the word "*eye*" of Yuanjiang was distinct from /t-/ in Southwestern Tai[40]. Despite the evidence, in this study, we provisionally adopted the classification of Xing[35] and Zhou and Luo[36] that Yuanjiang was classified in Southwestern Tai. Such classification was also adopted by the widely-used linguistic database of Glottolog (Glottocode: taih1246) and Ethnologue (ISO 639-3 code: tiz).

In summary, several fundamental questions on Kra-Dai language classifications deserved further examination based on more traditional linguistic comparative studies. Accordingly, it allowed us to obtain more linguistic materials from diverse linguistic perspectives such as

phonology and grammar.

1.4. Model settings in BEAST

We used BEAST v2.6.3 to infer language phylogeny and divergence time. BEAST v2.6.3 is a powerful tool with various models to perform Bayesian phylogenetic analysis on linguistic data. In this study, we used two substitution models, two molecular clock models, and two site heterogeneity models (Supplementary Data 3). The first substitution model is the continuous-time Markov chain model (CTMC)[41,42], which assumes that every language can gain or lose a cognate at a state-specific rate; the second substitution model is the binary covarion model[43,44], which allows the evolution rate of cognates to vary between a slow rate during periods of stability and a fast rate when bursts happen. Following our previous study[45], we did not apply the Pseudo-Dollo model[46,47] because this model assumes that once a cognate is gained, it could only be lost once and never be gained again, which did not intrinsically agree with the complicated and contact-frequent language evolution scenario in our Kra-Dai database. The first clock model is the strict clock model, which assumes that substitutions happen at the same speed across the whole tree with a specific rate parameter; the second is the uncorrelated relaxed model with a log-normal distribution, which permits the rate for each branch in the tree to vary within the given log-normal distribution. The two site heterogeneity models comply with the gamma distribution with one or four rate categories. These models allow rate variation across cognate sets and were applied for the CTMC model.

1.5. Settings of discrete phylogeographic inference

To estimate the transitions among different areas and reconstruct the ancestral area of Kra-Dai languages, we applied phylogenetic comparative approaches by using a reversible-jump Markov-Chain Monte Carlo approach (RJ-MCMC)[48]. The RJ-MCMC automatically evaluates which transition rates between states can be set to zero, and which rate parameters can be equal to the others[48]. The possibility of an ancestral area for each internal node is also estimated (Supplementary Data 4). The RJ-MCMC accesses the universe of *all possible* models of evolution visiting the different models in proportion to their likelihood. Specifically, in this study, we evaluated five different models for the regional transition of Kra-Dai languages (Figure S8). In particular, the FULL model allows state transition between every two regions even though they are not interconnected with each other geographically. Model 1 disallowed the transition between geographically no-adjacent areas, such as the transition between inland areas (Guizhou, Yunnan province) and Hainan Island, and the transition between the non-border area (Guizhou province) and MSEA. Model 1 is more reasonable for geographic inference in this study because the intermediate area is requisite for demographic transition. Derived from Model 1, the other models (Models 2, 3, and 4) further examine whether the transition between MSEA and one border area (Hainan Island, coastal area, Yunnan province, respectively) is disallowed (Figure S8). We used the Bayes Factor (BF), which is the ratio of posterior to prior odds, as the indicator for determining the optimal model. The RJ-MCMC was run 3 times to ensure the stability of the results (Supplementary

Data 10).

1.6. Mitochondrial DNA sample collection and raw data processing

Genomic DNA, extracted from blood samples, were sheared to 200-250 bp length, fixed to blunt-end, added with 3'-A tails, and ligated with barcode-linked Illumina paired-end adaptors. Then, ligation products were amplified by PCR, and quantified for pooling together. The mitochondrial DNA was enriched using custom designed bait. Finally, the pools were sequenced with HiSeq2000 sequencer.

Original sequencing reads were exported to Fastq files, and then bwa[49] was used to align reads to revised Cambridge Reference Sequence to generate binary sequence alignment/map (BAM) files of mtDNA genomes[50]. The duplicate reads were removed by MarkDuplicates, implemented in Picard (http://broadinstitute.github.io/picard/) and the mtDNA sequences were locally realigned by GATK[51]. Pileup files were generated by SAMtools[50]. Consensus sequences were then obtained based on the pileup files and indels were checked manually afterward. Additional published mtDNA genomes of Dong and Zhuang people were also included[52,53]. We also collected mtDNA genomes of Dai people from Yunnan Province of China, and Kra-Dai-speaking populations in Laos, Vietnam, and Thailand in the previous literature[54-57].

Complete sequences were aligned to rCRS by MUSCLE v3.8.31[58] and manually checked, and they were then assigned to haplogroups according to PhyloTree Build 17[59]. An mtDNA phylogeny was reconstructed using PhyML v3.1 with an HKY+G model[60], of which the main topology was consistent with that of PhyloTree Build 17. We identified the mtDNA lineages representing the expansion of the Kra-Dai languages based on the following principles. The mtDNA lineages should include at least four derived haplotypes which were composed of at least three regions of Kra-Dai-speaking populations and should include either Hunan Dong or Guangxi Zhuang people. Then, these lineages, usually star-like lineages, included considerable Kra-Dai-speaking individuals, representing the Kra-Dai-speaking population expansion, and range diffusion throughout different regions in East and Southeast Asia. In addition, we have provided detailed information on the mtDNA data in Supplementary Data 12, including sample names, accession codes, sources (whether newly generated or derived from published references), haplogroups, locations, population information, and selection status for BSP analysis.

1.7. Description of archaeological, paleoecologic, and paleoclimatic data

The time ranges and geographic locations of the archaeological sites were from the study of Hosner *et al.*[61] (URL: https://doi.pangaea.de/10.1594/PANGAEA.860072). Hosner's data contains a total of 51,074 archaeological sites from the early Neolithic to the early Iron Age (about 10,000 – 2,000 years before the present (BP)) with a spatial extent covering most regions of China. The information on each site included the cultural name (e.g., Liangzhu and Tanshishan cultures), time range (max, min, average), and geographical location (province, longitude, and latitude). Their data were integrated from three major campaigns of systematic archaeological surveys waged by the

Chinese government in 1956, 1981, and 2007. These data shed light on the spatiotemporal patterns of archaeological site distribution in China from the early Neolithic to the early Iron Age. We derived the available data from archaeological sites covering the geographic regions of Fujian, Guangdong, Hunan, Yunnan, and Zhejiang provinces from 9,000 to 2,000 years BP. These areas are located in the vast region of southern China where the present Kra-Dai-speaking populations lived. Accordingly, the chronological change in archaeological site numbers can be used as a proxy indicating the demographic background of Southern Chinese populations including the Kra-Dai-speaking populations.

The palaeoecological data were from the study of Gutaker *et al.*[62] (URL: https://doi.org/10.1038/s41477-020-0659-6). Gutaker's data contains the percent probability of tropical rice being in the thermal niche (assuming a requirement of 2,900 GDD at 10 °C base) during 5,500 – 1,000 years BP. These data were derived from their calculation by constructing a thermal-niche model[63], which estimates the probability of tropical rice cultivation in different areas. According to their work[62], they concluded that "*survival probabilities of tropical japonica between approximately 4,400 and 3,500 yr bp dropped substantially in eastern China and high-altitude southwestern China (survival probability<50%) compared with Southeast Asia (survival probability>90%)*". They profiled a scenario that the collapse of tropical rice cultivation caused by the 4.2k event[64] coincided with the southward migration of farmer communities. Accordingly, the paleoecologic data used in this study can approximately reflect the prehistoric agricultural development in South China and MSEA.

The paleoclimatic data were from the study of Fang *et al.*[65] and Hou *et al.*[66]. Fang and Hou's data contain the Holocene temperature series in China, with both the quantification and the higher temporal resolution continuously. They collected 1140 effective temperature records from previous references. Then, they reconstructed the Holocene temperature series in China with a synthesis reconstruction method, named the converted single sample from local to regional, and averaged it by the multiple samples. Their data could be regarded as good representative temperature series on a hemispherical scale. However, their study did not include the temperature records in MSEA. Therefore, the paleoclimatic data used in this study approximately reflect the changing trend of the global temperature in China.

1.8. Description of 4.2K event

The 4.2K event has been reported as a global cooling event that occurred around 4,200 years BP. Walker *et al.*[64] pointed out that the likely onset and termination of the 4.2k event might be 4,200 years BP and 3,900 years BP, respectively. Gutaker e*t al.*[62] provided a broader range that suggested the 4.2k event might start at 4,400 years BP and end at 3,500 years BP. Fang and Hou[65,66] did not give an accurate range for the 4.2k event but discovered several warm events, two of which occurred around 4,700 years BP and 3,500 years BP. In other words, these two points in time could be used as the upper and lower limit for the 4.2k event. Finally, we adopted the range used by Gutaker that the 4.2k event might start at 4,400 years BP and end at 3,500 years BP.

2. Supplementary Discussion

2.1. Disputations on the internal classifications of Kra-Dai languages

Kra-Dai languages consist of five well-known branches named Kra, Hlai, Ong-Be, Kam-Sui, and Tai[67]. However, the internal relationships among these five branches have been controversial for a long time.

The first controversy is the position of Kra. Most linguists are in favor of Kra as a primary branch in the Kra-Dai language phylum such as Liang and Zhang[2], Edmondson and Solnit[68], Diller[69], Chamberlin[70], Ostapirat[71], and Li[72]. However, Ostapirat[67] proposed an original bifurcation between the Northern and Southern groups at an early stage, which demoted Kra to the subbranch of the Northern group. This classification was further revised by Norquest[73], who demoted Kra to the position below the Kam-Sui group and as a sister of the Hlai-Tai branch.

The second controversy is the position of Hlai. Several scholars like Liang and Zhang[2], Edmondson and Solnit[68], Diller[69], and Chamberlin[70] asserted that Hlai is also a primary branch like Kra. Notably, all of them were in favor of a trifurcation structure for the initial divergence. Therefore, according to their opinions, both Kra and Hlai could be the primary branches of the Kra-Dai phylum. In contrast, Ostapirat[67] and Norquest[19] proposed that Hlai should be demoted to a sister of Be-Tai. Ostapirat[71] also suggested a bifurcation structure for the whole Kra-Dai phylum which places Hlai as the sister of a monophyletic group consisting of Ong-Be, Kam-Sui, and Tai.

The genealogical relationship of Ong-Be to other branches has not yet reached a consensus. Several linguistic scholars asserted that Ong-Be should fall outside of Tai but be closer to it than other branches (e.g., Hansell[74], Liang and Zhang[2], Edmondson and Solnit[68], and Norquest[19]). The Proto-Be-Tai was a daughter language of Proto-Kam-Tai whereas the other is Proto-Kam-Sui. However, Ostapirat[71] suggested that Ong-Be should be a sister to a monophyletic group consisting of Tai and Kam-Sui. The two daughter languages of Proto-Kam-Tai in a narrow sense were Proto-Kam-Sui and Proto-Tai.

In addition, debates on low-level branches of the Kra-Dai phylum remain ongoing. For example, there is a controversy about whether the division between the Central and Southwestern branches is on par with its Northern branch. Previous linguistic studies from Li[4], Liang and Zhang[2], Edmondson and Solnit[68], and Diller[69], etc. supported the tripartite schema for Tai languages which divided the Tai languages into three branches: Northern Tai, Central Tai, and Southwestern Tai. In contrast, other studies (e.g., Gedney[75], Haudricourt[76], Chamberlin[77], Strecker[78], and Ferlus[79]) suggested that the southwestern and Central languages should be placed into one group. Moreover, Edmondson[80] showed a much more diversified Central Tai phylogeny with computational phylogenetic analysis, suggesting that CT is not monophyletic and is split up into multiple branches, which was in agreement with the Pittayaporn's preliminary classification[6].

2.2. Phylogenetic topology of the Kra-Dai languages

Here, we performed a Bayesian phylogenetic analysis on 646 lexical cognates of 100 Kra-Dai

language samples to reconstruct the phylogenetic relationships among Kra-Dai languages. A moderately reliable phylogeny of the Kra-Dai languages was described by the best-fitting combination of the Covarion model with the Relaxed Lognormal clock model. The Bayesian phylogenetic tree showed five monophyletic groups of Kra, Hlai, Ong-Be, Kam-Sui, and Tai languages. Each of the five monophyletic groups possessed a posterior probability of 1, which strongly supported Ostapirat's classification [67,71]. Kra languages diverged from the proto-Kra-Dai language as the earliest branch indicating that the Kra language was the primary branch of the whole Kra-Dai languages. The other four branches of languages formed a monophyletic group with a posterior probability of 0.7. Our results supported the opinion of previous studies that Kra should be the primary branch in Kra-Dai languages (e.g., Liang and Zhang[2], Edmondson and Solnit[68], Diller[69], Chamberlin[70], and Ostapirat[71]).

The Hlai branch became the sister of a monophyletic group that consists of Ong-Be, Kam-Sui, and Tai languages. The rest three branches of languages formed a monophyletic group with a posterior probability of 0.95. Therefore, Hlai languages should be placed in the second diverged branch, which was consistent with Ostapirat's view [71].

The Ong-Be languages fell outside of the narrow sense of Kam-Tai languages, which only comprised Kam-Sui and Tai languages. Kam-Sui and Tai languages consisted of a monophyletic group with a posterior probability of 1, respectively. These branching patterns were in line with Ostapirat's view [71].

In contrast to the high-level relationships, the low-level branches showed a more complicated phylogenetic relationship that was not completely consistent with traditional linguists' expectations. In particular, the Kra languages majorly conformed to Ostapirat's view[21] which classified Buyang languages and Pubiao in a monophyletic group; while Gelao languages, Lachi and Laha in another. However, the Paha language (KraBuyangBH) was estimated to be more related to Lachi and Laha languages in our results, whereas Ostapirat suggested that the Paha language should be related to the Buyang-Pubiao group. The Hlai languages majorly conformed to Norquest's classification[19], which suggested that Cun and Nadou languages should be a sister group to Meifu dialects (HlaiChangjiang, HlaiXifang) and supported a monophyletic group of Qi dialects (HlaiBaoting, HlaiQiandui, and HlaiTongza). However, Run (Bendi) dialects (HlaiBaisha and HlaiYuanmen) were placed as a sister group of Qi dialects in our results but not a sister group of Meifu dialects in linguists' view. The Ong-Be languages were completely consistent with Ostapirat's view[81], which suggested that the Jizhao dialect branched first and the other Ong-Be languages were split into western and eastern groups. In contrast, in the Kam-Sui branch, we could not confidently determine the relationships among Mulam, Then, Mak, Jin, Maonan, and Chadong languages due to the low posterior values of the internal nodes. However, the place of Biao and Lakkia in our result supported Solnit's view[82] regarding Biao and Lakkia as a monophyletic group coordinated with Kam-Sui. Finally, the Tai languages were split into two parts roughly based on their locations. The Northern Tai languages were grouped with Yongnan dialects of Central Tai languages (TcFusui, TcShangsi, TcLongAn, TcQinzhou, and TcYongning), whereas the Southwestern Tai languages were grouped

with the other Central Tai languages. The inexistence of monophyletic Central Tai languages was advocated by Edmondson[68,80] and Pittayaporn[6,7]. In addition, we found that six Shan varieties (TswAiton TswHsipaw, TswTaunggyi, TswMangshi, TswMenglian, and TswKhuen)[36,83-85] collectively consisted of a paraphyletic group, a lower-level clade of Southwestern Tai languages. Despite other non-Shan varieties included in this clade, this node implied that a Proto-Shan language might yet be present. However, note that the low posterior value of this node (= 0.31) suggested that this internal node should not be robust in our results. In addition, the Saek language as a Northern Tai language seemed to be misplaced into the Southwestern Tai languages supported by a posterior value of 0.52.

There were some reasons for such misplacements observed in our phylogenetic tree. First, to give a straightforward display, we presented the maximum clade credibility (MCC) tree based on the posterior samples calculated from the MCMC method. The MCC tree could be interpreted as a global optimum tree for clade credibility. For a given node, a lower posterior value represented a less stable structure. Thus, we could not avoid the misplacements that might occur in several clades. Second, our work was solely relying on lexical cognates while most traditional linguistic classifications were based on both phonology and morphology. Third, the Swadesh 100-word list could not provide sufficient resolutions to distinguish the low-level relationships among Kra-Dai languages, especially when these languages experienced rapid differentiation in a short period and substantial language contacts (e.g., lexical borrowings) especially occurring during the period of initial language divergence. Fourth, the borrowing-prone languages were difficult to evaluate their linguistic relatedness, because it would be difficult to determine which linguistic traits were inherited from a common ancestor and which were borrowed from other languages.

Here, we took Saek as an example. Saek is a minority language of Northern Tai in Thailand but is the substantial contact-induced change from its surrounding Southwestern Tai languages (e.g., Thai and Lao languages)[86,87]. To explore which reason has led to the misplacement of Saek and address the authentic genealogical classification of Saek, Northern Tai, and Southwestern Tai, we performed the four-point analysis which could provide the possibilities of a specific two-to-two partition directly estimated from the linguistic data given the sub-tree structure. As this method examined all the cognate sets one by one, the possibility for a given structure could be considered to be the proportion of potential cognate sets (inheriting from a common ancestor or borrowing from other languages) in the tested language and its nearest language in the given structure[88]. Following the computational procedure in our previous study[88], the results of the four-point analysis showed that the possibility for (Saek, Tsw)-(Tn, Others) was 0.3716, for (Saek, Tn)-(Tsw, Others) was 0.6275, and (Saek, Others)-(Tn, Tsw) was 9.2593×10^{-4}. The subtree structure for (Saek, Tn)-(Tsw, Others) was moderately supported, indicating that Saek should belong to the Northern Tai group rather than the Southwestern Tai. However, the subtree structure for (Saek, Tsw)-(Tn, Others) was weakly supported, indicating that Saek might have considerable borrowings (37.16%) from Southwestern Tai languages. Such a vast number exceeded the 20% limit of the Bayesian phylogenetic methods for borrowing words[89], which could potentially twist the tree structure.

Therefore, we suggested that the given lexical cognate data were sufficient to distinguish the fine-scale relationship among similar languages under given circumstances. The misplacement of Saek in the Bayesian phylogenetic tree resulted from methodological inadaptability for distinguishing borrowing-prone languages. This was also supported by linguistic views that Saek could experience substantial borrowings or replacements from its surrounding Southwestern Tai languages [86,87].

In summary, our reconstruction of the phylogeny of Kra-Dai languages mostly conformed to previous linguists' views, especially in the high-level branches. However, we could also observe several misplacements due to the deficiencies of Bayesian phylogenetic methods (more discussion regarding these methods see the section "*Bayesian phylolinguistics, proper-used or misused?*" below).

2.3. The divergence time of the Kra-Dai languages

After calibrating several internal nodes (Supplementary Data 2), we estimated the divergence times of all internal nodes on the Kra-Dai phylogenetic tree. The estimations for the average root ages were approximately 4,000 years BP, and they were compatible with each other in all the tested model combinations (Figure S6 and Supplementary method). In the best-fitting model with the maximum marginal likelihood (Supplementary Data 3), the average time estimation for the initial divergence of the Kra-Dai languages was 4,041 years BP (95% HPD: 2,741 - 5,550 years BP). The MCC tree with node bars of 95% HPD denoted on all internal nodes was also shown (Figure S3). Notably, we estimated the average divergence time of Proto-Kra to be 2,435 years BP (95% HPD: 1,967 - 2,909 years BP); the estimated average divergence time of Proto-Hlai was 1,155 years BP (95% HPD: 443 - 2,035 years BP); the estimated average divergence time of Proto-Ong-Be was 1,750 years BP (95% HPD: 1,299 - 2,226 years BP); the estimated average divergence time of Proto-Kam-Sui was 1,222 years BP (95% HPD: 1,044 - 1,410 years BP); the estimated average divergence time of Proto-Tai was 1,360 years BP (95% HPD: 873 - 1,903 years BP) (Figure 1c).

2.4. Discrete phylogeographic inference

For the NULL hypothesis of the phylogeographic model, we could observe 20% for each distinct area which was inferred as a dispersal center equiprobably. Here, our phylogeographic reconstructions indicated that the most likely dispersal center of Kra-Dai languages was in the coastal areas of China with a maximum probability of 47.0%, which was much higher than 20%, as shown in Supplementary Data 4. In addition, we used the paired one-side Wilcoxon signed rank test to find that the probability of the coastal area was significantly higher than those of the other four distinct areas. Accordingly, we suggested that the coastal area should be the homeland of Kra-Dai languages with a significantly higher probability than other areas (Figure S7). Meanwhile, our analysis suggested that the Proto-Kra language diverged from the Proto-Kra-Dai language around 4,000 years BP and spread northwestward into inland South China, specifically Yunnan and Guizhou provinces. The Proto-Hlai language diverged later and spread to Hainan Island at approximately 3,200 years BP, followed by the origin of the Proto-Ong-Be language around 2,600 years BP, which

also spread to Hainan Island. Notably, the "into the island" scenario was supported by the transition rates estimated in this study, while the "out of the island and back to the mainland" scenario occurred less frequently. The Proto-Kam-Sui and Proto-Tai languages likely remained in the coastal area for a considerable period until around 1,300 years BP. Then, the Kam-Sui languages might have spread northwestward to mountainous Guizhou, while the Tai languages spread throughout the vast region of South China and MSEA. Additionally, the transition rates suggested that most of the Kra-Dai languages in MSEA might have first reached Yunnan province and then spread southwestward into MSEA, while only a small portion might have spread directly from the coastal area (Figure S9).

In conclusion, the Coastal Origin Hypothesis is strongly supported by our analysis, indicating that the most likely dispersal center for the Kra-Dai languages was coastal South China (Figure S1b and Figure S7. The dispersal routes of the Kra-Dai languages followed an "out of China" scenario[90], with higher transition rates from China to MSEA than in the inverse direction. Moreover, we identified north-south and east-west dispersal routes for the Kra-Dai languages [72] (Figure 2, Figure S8, and Figure S9), which also reflect the history of human population migration since languages are carried by people [91].

2.5. Bayesian phylolinguistics, proper-used or misused?

Recent advances in Bayesian phylogenetic methods from evolutionary biology provide alternative opportunities to permit flexible evolutionary models to reconstruct more reliable genealogical relationships. Over the last two decades, computational linguists have incorporated these methods into linguistic research and made significant progress in reconstructing the prehistories of well-known language families worldwide[42,88,92-95]. Consequently, Bayesian phylolinguistic methods that employ Bayesian phylogenetic methods to evaluate linguistic datasets and reconstruct language phylogenies have become a potent tool for inferring the tempo and mode of change in language families.

However, Pereltsvaig and Lewis[96] raised several specific critical comments for the flaws of Bayesian phylolinguisitc methods. In particular, we summarized these flaws as three major issues: (1) examining only lexical material; (2) inadequately identifying borrowings; (3) ignoring the misplacement of individual languages on the family tree.

Regarding issue (1), different language subsystems should experience distinct evolutionary processes from the past to the present. For example, phonology and lexicon exhibit different evolutionary patterns[97], and linguistic features evolve at various rates[98]. Furthermore, there are varying degrees of horizontal influence on phonological, grammatical, and lexical subsystems[99]. As a result, investigating language relationships using different linguistic features could lead to different classifications due to these evolutionary processes. However, grammatical, phonological, and even phonetic traits may not be suitable for dating as they tend to vary more freely and rapidly than core vocabulary items[98]. Additionally, cognate judgments of lexical data involve phonological and morphological knowledge[100]. Moreover, lexical data is universally available and can be identified in a large number of cognate sets in lexical meanings[100]. In contrast, grammar, phonology,

and morphology offer limited data characters and can only define a few controversial subgroups within a family, which hardly contribute to higher-order subgrouping[100-102]. Therefore, we suggested that lexical data remained crucial for establishing linguistic relatedness because of the stability, comprehensiveness, availability, and size advantage.

Regarding issue (2), we acknowledged that even the core lexicon could be borrowed during language contact. In practice, we have made every effort to identify definite borrowings in Kra-Dai languages, but it was possible that a small number of undetectable borrowings still existed. Fortunately, computational simulations of phylogenetic methods in several previous studies have given us confidence that without any linguistic constraint on the phylogenetic reconstruction, the number of undetected borrowings would need to be substantial (>20%) to significantly bias either the tree topology or date estimates[89,92]. Additionally, the tree structure emphasizes the vertical process of language diversification rather than horizontal contacts and admixture. The extent of language contact can be measured by delta score and Q residual value, which were discussed in the section "*The homoplasy in the Kra-Dai language phylogeny*" in the Supplementary Information.

Regarding issue (3), it should be noted that the phylogenetic tree is only a hypothetical representation of language diversification, and its topology is dependent on the input data. The uncertainty of the topology is shown by the posterior value of each internal node, where higher values indicate more robust evidence for grouping downstream languages as a monophyletic group. In our study, the maximum clade credibility (MCC) tree was used to represent the relationships among Kra-Dai languages, which is interpreted as the global optimum tree. However, it was possible that some internal nodes in the MCC tree did not appear in other posterior samples, suggesting that some languages would be monophyletic in the MCC tree but paraphyletic in reality. These uncertainties are represented by the web structure in DensiTree[103] (Figure S4). The posterior values of higher-order internal nodes in our MCC tree were mostly higher than 0.9, indicating strong support for the relationship of the five major branches of Kra-Dai languages.

In our study, we indeed observed low posterior values and misplacements for several low-level internal nodes (mentioned in the section "*Phylogenetic topology of the Kra-Dai languages*" before). The Swadesh 100-word list, for example, may not provide sufficient resolution to distinguish low-level relationships among similar languages within a whole language phylum, especially when they have experienced rapid differentiation in a short recent period and substantial language contacts. Moreover, traits derived from horizontal language influence could be another factor affecting the robustness of the phylogenetic structures of languages. Additionally, as mentioned in issue (1), grammatical, phonological, morphological, and other linguistic features, which could be effective in distinguishing similar languages, were mostly excluded from the reconstruction of phylogenetic trees. These limitations could result in minor misplacements of individual languages, especially at the low-level branches. Therefore, to reconstruct a more robust and fine-scale structure for low-level branches, it was necessary to collect more comprehensive and detailed linguistic data and to improve Bayesian phylolinguistic methods to account for the heterogeneity of various linguistic traits.

Meanwhile, these misplacements would further challenge the robustness of the whole language phylogenetic tree. Fortunately, a previous study indicated that minor misplacements of individual languages at lower levels would not affect the high-level relationships among language branches, nor the overall shape of the tree[104]. To test whether these minor misplacements would impact our main results on linguistic relatedness of the five language branches, time depth, and dispersal center, we conducted four different settings during the reconstruction of language trees: (1) default settings (version in the manuscript); (2) constraining the languages of the same groups as monophyletic groups, respectively (i.e., Kra, Hlai, Ong-Be, Kam-Sui, Southwestern Tai, Central Tai, and Northern Tai); (3) excluding the Saek language from our data; and (4) constraining the six varieties of the Shan language as a monophyletic group. The first setting had no prior constrains and was the version used in this study. The second setting ensured Kra, Hlai, Ong-Be, Kam-Sui branches, and the three Tai groups to be monophyletic respectively and constraining Saek into the Northern Tai group according to traditional linguists' views[4,75,86,87]. The third setting excluded Saek because it was suggested as a borrowing-prone language that would undermine the overall shape of the tree[42,86,87,104]. The fourth setting ensured the presence of Proto-Shan by constraining the Shan varieties[36,83-85]. Since Shan populations were influential in MSEA[84,105], the varieties of Shan language would experience substantial contacts with other languages and failed to form a monophyletic group. These four different settings would generate four sets of trees with different low-level branching patterns, which would be then used for further analysis to compare linguistic relatedness, time-depth, and dispersal center. The model used for tree reconstruction was a combination of the Covarion model and the Relaxed Lognormal clock model, which was the best-fitting one under default settings. The studies for discrete phylogeographic inference were also consistent with those in the section "*Discrete phylogeographic inference*" in Methods. The results were illustrated in Figure S11, Figure S12, and Supplementary Data 5. All results supported Ostapirat's linguistic relatedness hypothesis[71]; agreed on the time depth (around 4,000 years BP), as well as other high-level internal nodes; and suggested that the coastal area was most likely to be the dispersal center of Kra-Dai languages. Therefore, our replications confirmed the robustness of our main conclusions on Kra-Dai languages using Bayesian phylolinguistic methods.

In summary, Bayesian phylogenetic methods are a powerful tool that can supplement traditional linguistic scholarship but not replace it. As a cutting-edge and promising computational approach, Bayesian phylogenetic analysis and other developing methodological variants (e.g., Neureiter *et al.*, 2022[106]; Koile *et al.*, 2022[107]) can shed light on investigating vertical transmissions, including the traditional task of reconstructing linguistic classification. It is crucial to continue the amelioration of these methods and to integrate them with traditional linguistic analysis to achieve a more comprehensive understanding of language evolution and diversity.

2.6. Genetic evidence of Kra-Dai population expansion

To investigate the genetic evidence of Kra-Dai language expansion, we inferred the demographic history reconstructed by the mitochondrial DNA (mtDNA) sequences of Kra-Dai-

speaking people with a Bayesian Skyline plot (BSP) (Figure S14). We found two expansion phases of these Kra-Dai representative lineages, of which the former was an approximately 17-fold demographic increase during 6,400 – 4,200 years BP and the latter was also an approximately 16-fold demographic leap from 3,500 years BP till now. Genetic evidence for Kra-Dai expansion in the late Holocene could be temporally aligned with the evidence from other disciplines.

2.7. The prehistoric cultures in coastal Southeast China ~ 5,000 years ago

To shed light on the demographic activities in coastal Southeast China around 5,000 years ago, we have synthesized interdisciplinary evidence from climate, agriculture, and genetics (Figure 2). Generally, the early rice farmers were the indigenous people in Lower Yangtze Valley, where rice domestication occurred before 6,000 years BP[108-110]. Although mixed farming was also present, the primary focus of labor was rice cultivation[111,112]. Paleoclimatic data revealed that the global temperature decreased with fluctuation between 6,000 and 4,400 years BP[66], making it reasonable for people to migrate towards warmer regions. The contemporaneous global sea-level rise of ~3m might have destroyed coastal settlements and further facilitated migration[113]. Then, coastal South China was an appropriate destination for the rice-based mixed farming people living in Lower Yangtze Valley, where they might have migrated southwards along the coastal line with their crops[114,115]. Ethnologists and archaeologists suggest that these Lower Yangtze Valley farmers were the common ancestry of both Kra-Dai and Austronesian people, based on shared culture[116,117], archaeological materials[118], and ancient DNA studies[119-121]. The cool climate between 6,000 and 4,400 years BP might have continuously compelled the southward migration, leading to the separation of the rice-based mixed farming peoples into two different populations. During this period, people who migrated along the coastal line on land became the ancestry of Kra-Dai speakers, while those who adopted a maritime lifestyle became the ancestry of Austronesian speakers[57].

According to ethnological and archaeological views[122,123], the Kra-Dai-Austronesian multi-ethnic populations can be traced back to the people of the Hemudu culture (7,000 – 5,300 years BP) and the Majiabang culture (7,000 – 6,000 years BP). These ethnic groups were named Bai Yue by ethnologists, who regarded Kra-Dai-Austronesian people as their descendants[124,125]. In ancient China, the term "Bai Yue" referred to the "hundreds of tribes", which were collectively known as ancient indigenous Kra-Dai-speaking populations living in present-day coastal southeast China[122,126]. A recent genetic study confirmed that Bai Yue ancestry was widely distributed in Kra-Dai-speaking populations in South China and MSEA.[127]. Their study found that although other genetic components were present, the Bai Yue lineage was dominant in contemporary Kra-Dai-speaking populations. In other words, the diverse present-day Kra-Dai-speaking populations descended from their common ancestor, the ancient Bai Yue lineage, which underwent different migration, admixture, and isolation over time.

In summary, we have presented a scenario for demographic activities in coastal Southeast China between 6,000 and 4,400 years BP (Figure 2). The Bai Yue lineage of rice-based mixed farmers in the Lower Yangtze Valley migrated southward with their crops along the coastal line,

eventually splitting into two populations: the Kra-Dai people and the Austronesian people.

2.8. The history of the Kra-Dai-speaking populations in the past 4,400 years

The ethnolinguistic diversity in Southeast Asia obscures the history of the region's nationalities and languages. Since human population activities are closely linked to language divergence, studying linguistic evolution can offer new insights into human history[91]. This study focused on the history of the Kra-Dai people over the past 4,400 years, which could be divided into two distinct periods. The first period occurred before the Qin Dynasty (4,400-2,300 years BP) while the second period took place after the Qin Dynasty (2,300 years BP to present), with the latter period being defined by the Han people's political domination of South China. Based on the interdisciplinary alignment, we proposed a plausible historical scenario for the Kra-Dai people in these two periods, as illustrated in Figure 3 and Figure S10.

In the first period (4,400 - 2,300 years BP, which was also referred to as the "contraction period" and the "recovery period"), there were dramatic fluctuations in the climate that led to the collapse of agriculture. This, along with increasing cultural pressure in the form of competition for natural resources (such as copper and pastoral grounds) and even the spread of plague epidemics, indicated that the semi-sedentary agro-pastoral populations had to expand to more suitable habitats[128,129]. Our study supported this scenario, as evidenced by the number of archaeological sites and the effective maternal population size. The number of archaeological sites first decreased due to the contraction and migration, and then increased due to the population expansion. Meanwhile, the population size continued to grow steadily. These demographic changes indicated that people were likely to migrate first and then settle down during this period, which further contributed to a discontinuous language divergence pattern (Figure S10). Specifically, we observed that the Hlai and Ong-Be languages, spoken by the migrants from the mainland to Hainan Island, successively split from the Kam-Tai languages during this period. Notably, interactions between the Han and Kra-Dai peoples were weak because the Han people's political domination was limited to the Middle and Lower Yellow River basin during the Xia, Shang, and Zhou Dynasties[122]. Therefore, we concluded that climate was the primary factor that drove the migration and settlement of the Kra-Dai people during the first period.

In the second period (2,300 years BP to the present, which was also referred to as the "prosperity period"), the environmental context, including climate and agriculture, was relatively more stable than that in the first period. However, the effective population size increased considerably, and language diverged rapidly. These results suggested that population interactions might have been the dominant factor shaping the history of the Kra-Dai people in the second period. Our conclusion was supported by historical records of the Kra-Dai people provided by Chinese scholars. The interactions between the Kra-Dai people and the central Chinese people greatly intensified since the Qin Dynasty[124]. The southward expansion of political power not only accelerated the assimilation of the Kra-Dai people but also impelled their emigration, such as the migration to the Yunnan-Guizhou Plateau or even out of China[2,34]. For example, during the Qin Dynasty, a war was waged to conquer Bai Yue after unifying six states (around 2,200 years BP)[2].

The war forced many Bai Yue populations to migrate, which later resulted in the increasing divergence rate (interval 1 in Figure S10) and the divergence of Kam-Tai languages (around 1,950 years BP). Subsequently, the second migration wave occurred in the Tang Dynasty, when the Zhuang ancestors resisted the reign of the Tang Dynasty for nearly one hundred years (around 1,200 years BP)[2]. However, the rebels of Zhuang ancestors were suppressed and could only migrate to MSEA, resulting in a rapid divergence in this period (interval 2 in Figure S10). This was followed by the divergence of the Southwestern Tai languages and their sister group of Central Tai languages (around 1,179 years BP). During the Song Dynasty (around 950 years BP)[2], the third migration occurred as a result of the failure of the uprising led by minority leader Nungz Cigauh. This led to another large-scale migration southwestward into MSEA, which coincided with a rapid increase in the language divergence rate (interval 3 in Figure S10) and the divergence of Southwestern Tai languages (around 824 years BP). This migration might have contributed to the unification of Thailand in the following decades. Our analysis, combined with reliable historical documents, suggested that political power was a major influence on the demographic activities of the Kra-Dai people during the second period.

In summary, we provided a brief overview of the history of the Kra-Dai people and proposed that environmental factors were a major driving force in the first period, while political power played an alternative significant role in shaping human activity in the second period.

2.9. Language/Farming Dispersal Hypothesis for Kra-Dai languages

The language/farming dispersal hypothesis has been proposed to explain the worldwide distribution of languages[130,131]. According to this hypothesis, prehistoric population expansions may have led to the spread of agriculture and languages to other areas.

In our study, we found evidence to support the idea that the dispersal of Kra-Dai languages was linked to the spread of agriculture in prehistoric times. According to previous studies[114,115], the Kra-Dai people were the descendants of rice-based farmers who initially settled in the Lower Yangtze Valley and then migrated southward along the coast, spreading both rice-based agriculture and millet farming[114,115,132,133] (Figure S13). Our analysis also revealed that the early dispersal center of Kra-Dai languages was in coastal South China (Figure 2 and Supplementary Data 4). However, in contrast to the common view that the language and agriculture expansion was driven by increased population size, high agricultural yields, and suitable ecological niches for farming[130], the language/farming dispersal of the Kra-Dai languages conformed to another scenario. According to our interdisciplinary analysis, the early-stage divergence of Kra-Dai languages coincided with the 4.2K event. During this event, we could find dramatic changes in the number of archaeological sites, a slowly growing population size, decreasing survival probability of rice, and climate fluctuation (Figure 3). These indicated that Kra-Dai people were impelled to migrate to find suitable land for farming due to climate change and agricultural recession. Therefore, we concluded that the prehistoric dispersal of Kra-Dai languages and agriculture in South China conformed to the language/farming dispersal hypothesis. Importantly, the driving force behind this dispersal appeared

to be the collapse of agriculture caused by paleoenvironmental change.

In contrast, the language/farming dispersal hypothesis might not apply to the early history of the Kra-Dai people in MSEA. The archaeological evidence suggested that the earliest agriculture records could be traced back to earlier than 4,000 years ago[132,134] (Supplementary Data 7). However, the initial Kra-Dai language diverged around 4,000 years BP in South China, and the spread of Kra-Dai languages in MSEA might have happened within the last 2,000 years. This inconsistency did not support the idea that the dispersal of Kra-Dai languages could be related to agricultural dispersal in MSEA. Furthermore, genetic evidence strongly suggested that the (Proto-) Austroasiatic-speaking migrants from south China were likely the ones who introduced agricultural innovations to MSEA[114,135]. Therefore, the farming/language dispersal hypothesis might not be applicable to explain the co-dispersal of agriculture and Kra-Dai languages in MSEA.

Nevertheless, it is essential to consider the impact of coastal expansion on agricultural development in MSEA. In coastal Southeast Asia, evidence of rice and millet plant remains has been found dating back over 4,000 years ago, predating those found in inland Southeast Asia[134]. This suggested that the rice and millet dispersal in MSEA might not have occurred solely through the north-south inland dispersal route but also through a possible maritime route originating from coastal South China[136,137]. This prehistoric marine trade network, which surrounded the South China Sea, was supported by abundant excavated material remains[102,138]. Since the genetic component of Kra-Dai people in MSEA was only observed in the past 2,000 years[139], it seemed that the early maritime route of agricultural dispersal was driven by cultural communication rather than demic diffusion. However, we could not rule out the possibility that cultural interaction also occurred in MSEA and South China through the inland or coastal route. In that case, we must re-evaluate the language/farming dispersal pattern in MSEA: cultural interaction could also have promoted the dispersal of agriculture without demic diffusion and language dispersal.

To summarize, we have examined the farming/language dispersal hypothesis of Kra-Dai languages and proposed a scenario for the dispersal of agriculture, the ancestral Kra-Dai people, and their languages from coastal South China to MSEA from a macroevolutionary perspective.

2.10. The prehistoric agricultural strategies in South China and MSEA

As previously discussed, the Kra-Dai people played a significant role in the agricultural development of South China and MSEA through their demographic activities. They likely introduced both millet and rice to these regions. However, when agricultural populations migrate to new environments, they must maintain a productive agricultural system in a new place, which leads them to adopt new and sustainable agricultural strategies[133]. As shown in Supplementary Data 7 and Figure S13, despite similar crop varieties (*japonica* rice and Asian millets) in South China and MSEA, there were significant differences between the archaeobotanical assemblages found at sites in both areas during the prehistoric period. It appeared that populations living in relatively high latitude, high elevation, and hilly dryland areas of South China cultivated more risk-oriented millet crops than those living in MSEA[136,140,141]. In contrast, populations living in the relatively low

latitudes with a wetland environment of MSEA opted for rice-based cultivation[136,140]. This suggested that temperature, latitude, altitude, and water supply could be the most critical factors that determine human agricultural strategies.

2.11. The genetic and cultural admixture patterns of Kra-Dai and surrounding populations

The history of Kra-Dai-speaking populations and their language culture is far from clear. Since Kra-Dai populations lived at the crossroads where five main language families have spread and diversified[142], Kra-Dai populations could not be simply modeled as inheriting directly from Bai Yue lineage and culture.

In South China, Kra-Dai populations have experienced substantial contact with the aboriginal Hmong-Mien populations deeply from the beginning of the late Holocene[115,143]. Specifically, for several geographically close ethnic groups of two language families, extensive genetic admixture was observed, and no clear genetic barrier existed among them[144,145]. These admixture scenarios formed a "Hmong-Mien Cline" showing that Hmong-Mien-speaking individuals from west to east roughly have a decreased proportion of Hmong-Mien-related ancestry component and an increased proportion of Kra-Dai-related ancestry component[146]. The Tibeto-Burman populations were another ethnic group that came to South China in the late Holocene. In contrast to the Hmong-Mien people, however, Tibeto-Burman people contributed little genetic influence to Kra-Dai people[143,144]. Moreover, Sinitic populations created powerful empires and expanded their political and military influences to South China in the last 2,000 years[114]. Their dominant political power has greatly contributed to the population admixture in South China[127,143]. In addition, Sinitic people also dominated cultural admixture and shift. These could be reflected in the presence of loanwords[69], variations in phonetic structures[34], and grammatical system[2]. Therefore, the admixture history of Kra-Dai-speaking populations in South China was related to frequent gene flow and their cultural communications with surrounding people, especially Hmong-Mien and Sinitic populations.

In MSEA, Austroasiatic-speaking populations were the main local people whereas Kra-Dai-speaking populations expanded to this region in the last 2,000 years[139,142]. The genetic study revealed that heterogeneity in admixture with local Austroasiatic groups and geographic proximity primarily shaped the genetic structure of Kra-Dai people[147]. In addition, Sino-Tibetan, Hmong-Mien, and Austronesian groups which also migrated to MSEA contributed limited genetic ancestry to Kra-Dai people[142,147]. Therefore, the extensive contact between the groups of different language families resulted in cultural diffusion and even a cultural shift in MSEA[148].

In summary, extensive contact with surrounding populations collectively shaped present-day Kra-Dai-speaking populations and their languages. Meanwhile, we expected more detailed studies to shed further light on their complex history.

2.12. The homoplasy in the Kra-Dai language phylogeny

To measure the extent of homoplasy or horizontal influence (e.g. lexical borrowing) which is

against the tree-like topology, we calculated the delta score[149] and Q-residual[150] value for each language sample. We performed the calculation in SPLITSTREE v4.17.1 (http://www.splitstree.org/) using Gene Content distance[151]. Higher values of Delta score and Q-residual indicate a higher degree of reticulation[150]. As shown in Supplementary Data 6, the delta score of the Kra-Dai languages was the lowest among all the languages. The value of the delta score is significantly correlated with the linguistic isolation of individual languages within their respective phylogenies[152]. This was consistent with the high support of the internal nodes of the five well-established branches in the Kra-Dai language phylum. According to our inferred history of the Kra-Dai people, linguistic isolation could result from their early geographical isolation and population migration[150]. Accordingly, we could observe a tree-like structure for Kra-Dai languages based on the delta score. In contrast, the Q-residual score of Kra-Dai languages was the third highest in the column. Since the Q-residual score is sensitive to the age of language phyla[152], we should compare the Q-residual score among the language phyla with the similar range of divergence time of other languages such as Dravidian languages. Among the seven language phyla, the divergence time of Dravidian languages was estimated as the closest one to that of Kra-Dai languages[45]. The Q-residual value of Kra-Dai languages was slightly higher than that of Dravidian languages. In contrast to the evolution of Dravidian languages, we thus speculated that Kra-Dai languages could have experienced more considerable potential horizontal influence, as well as the formation and break-up of dialect chains[150] in the low-level branches. In addition, the low-level branches were mainly contemporaneous with the appearance of an international trade network in East Asia[147]. This suggested that the core lexical items might change with the borrowing of trade terms and then a high Q-residual was observed. This was also in accordance with the linguistic ecology that the present Kra-Dai languages are distributed in the extensive ethnolinguistic regions surrounded by Sino-Tibetan, Hmong-Mien, Austronesian, and Austroasiatic languages.

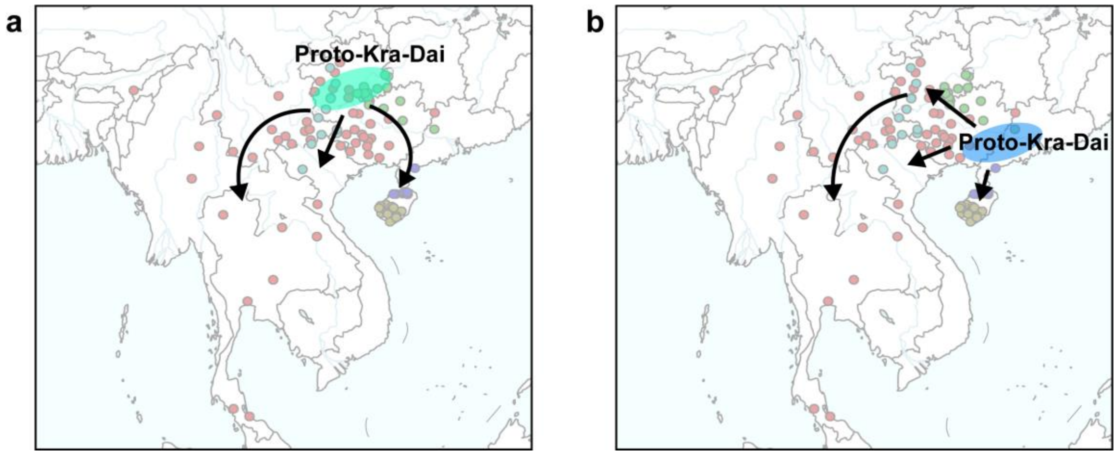

Figure S1. The two possible dispersal routes of Kra-Dai languages. (**a**) the dispersal routes of Inland Origin Hypothesis. The green oval is the possible dispersal center of Proto-Kra-Dai. (**b**) the dispersal routes of Coastal Origin Hypothesis. The blue oval is the possible dispersal center of Proto-Kra-Dai. The colored small dots are the geographical locations of language samples used in our study. The base maps were derived from an R package *rnaturalearth* (URL: https://github.com/ropensci/rnaturalearth).

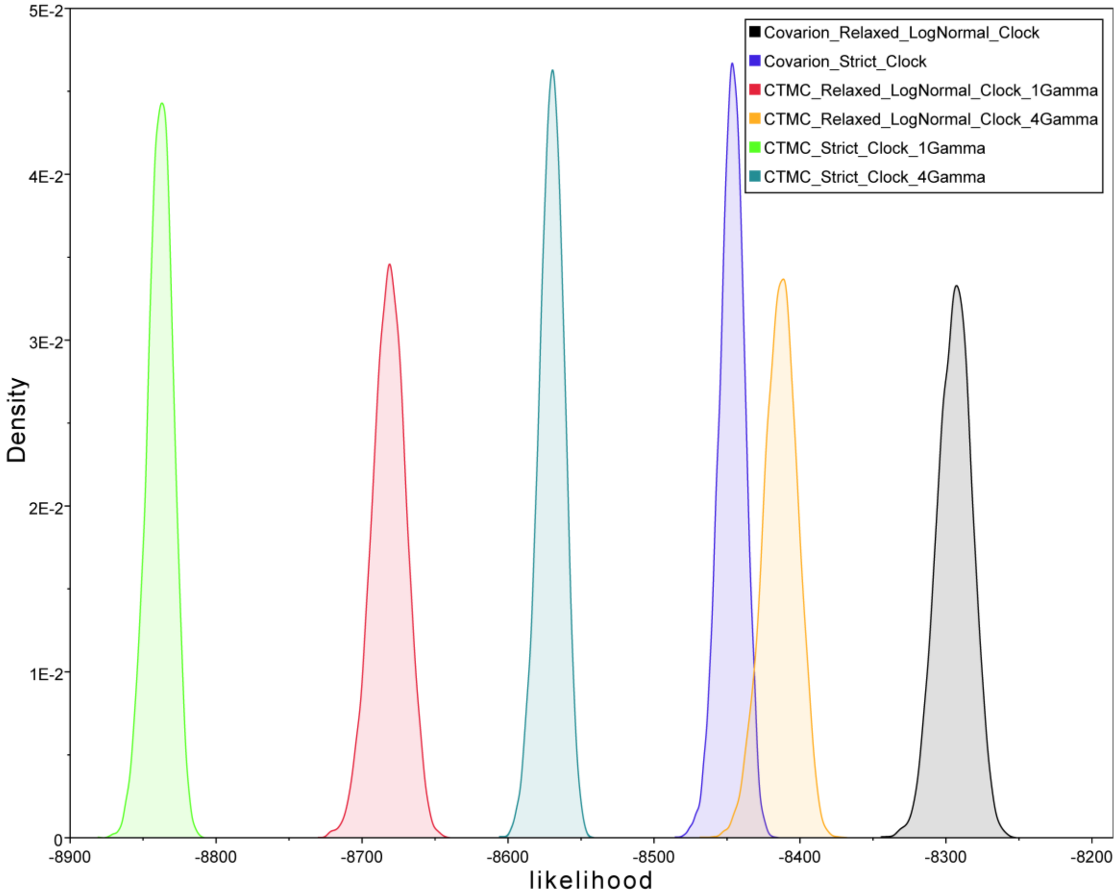

Figure S2. The distributions of the likelihood values for six models. The likelihood for Kra-Dai languages under six combinations of models. Each model was run for 50,000,000 generations, in a sampling frequency of 5,000, with a burn-in of the first 10% of samples.

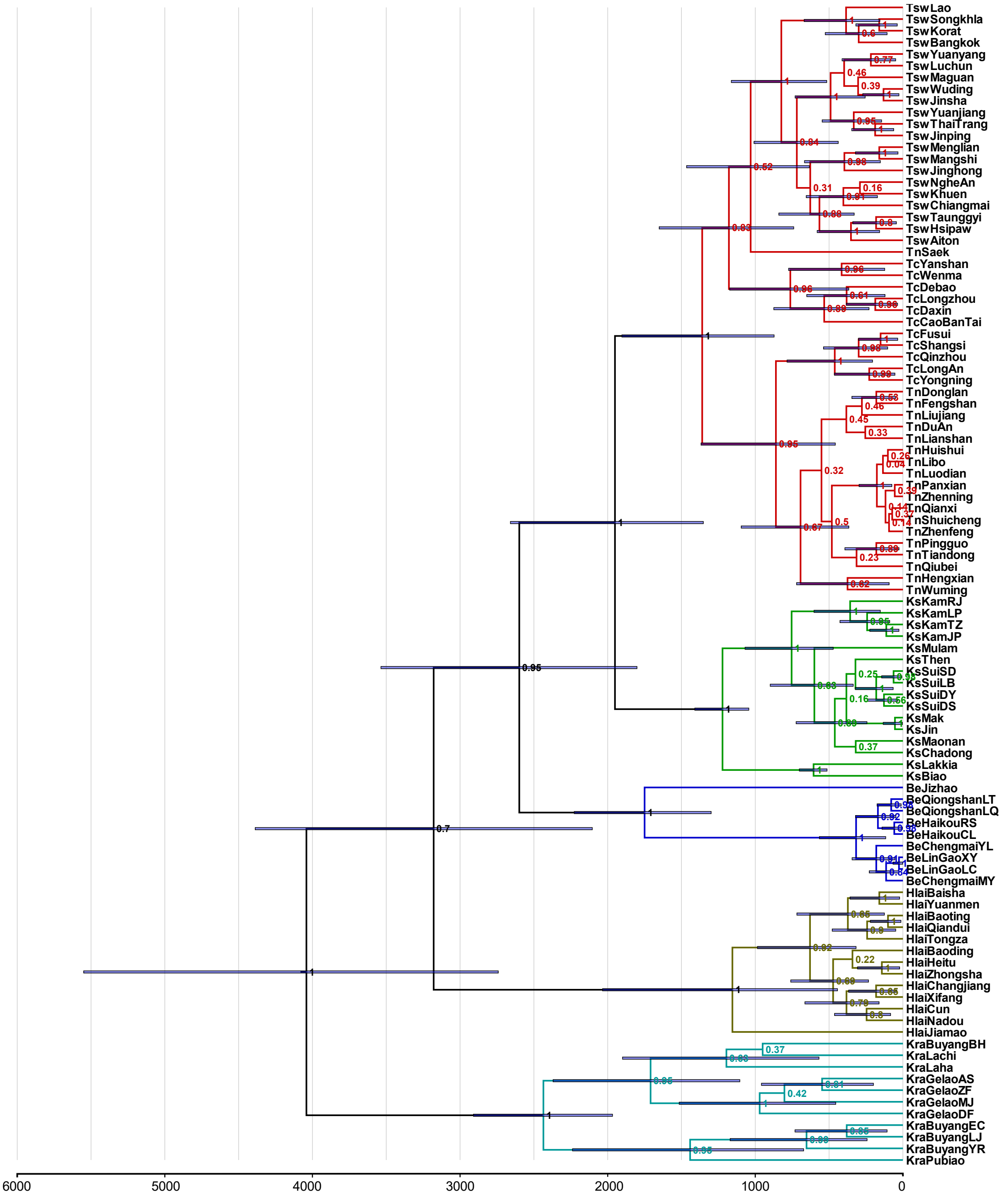

Figure S3. The maximum clade credibility tree of 100 Kra-Dai languages with node bars of ages and posterior probability values. The reconstruction of the MCC tree, as well as the estimation of node bars of ages of 95% HPD and posterior probability values, were based on Covarion + Relaxed LogNormal clock model. This model was run for 50,000,000 generations, in a sampling frequency of 5,000, with a burn-in of the first 10% of samples.

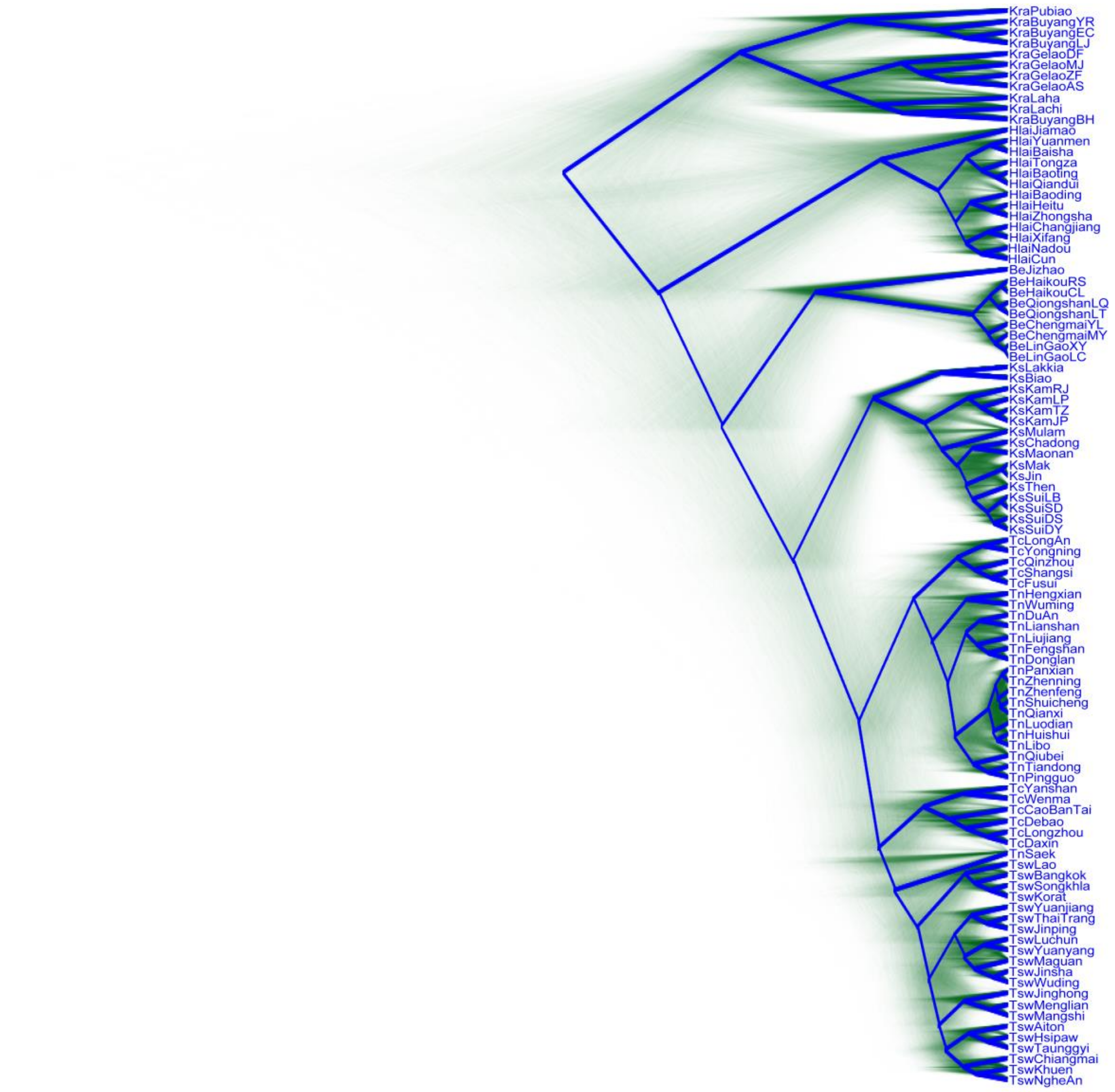

Figure S4. The DensiTree of 100 Kra-Dai language. The DensiTree is generated by the DENSITREE v2.2.7 program based on the results of the Covarion + Relaxed Lognormal clock model. Highly colored areas were the consistent topology and branch lengths of posterior trees; whereas webs were areas with little agreement.

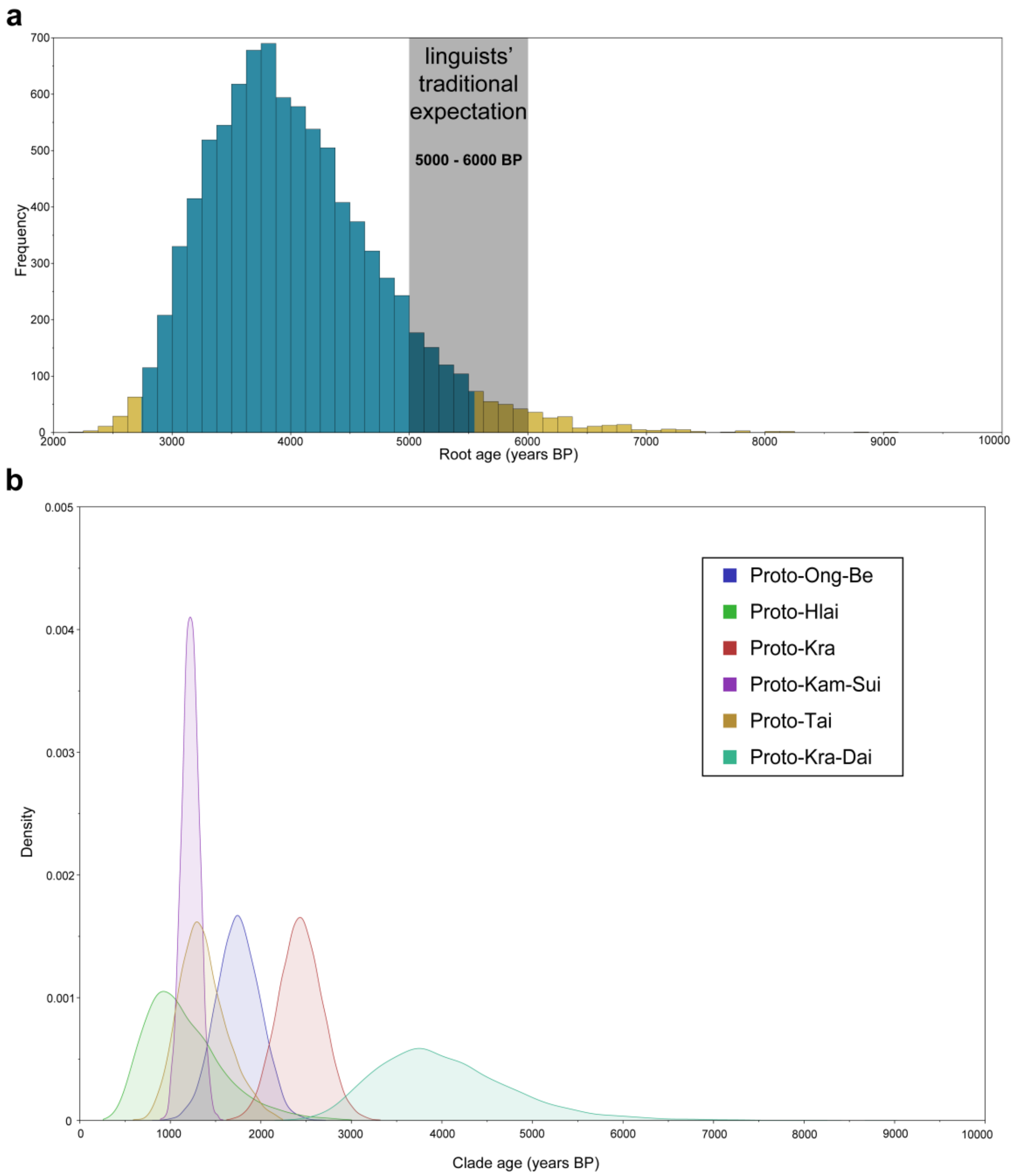

Figure S5. The clade ages of Proto-languages under Covarion + Relaxed Lognormal clock model. (**a**) The histogram plot of root (Proto-Kra-Dai) age. The shaded grey part indicates the predicted root age for the Proto-Kra-Dai language of the traditional mainstream view (5,000-6,000 years BP)[2,72]. (**b**) The distribution of ages of Proto-languages.

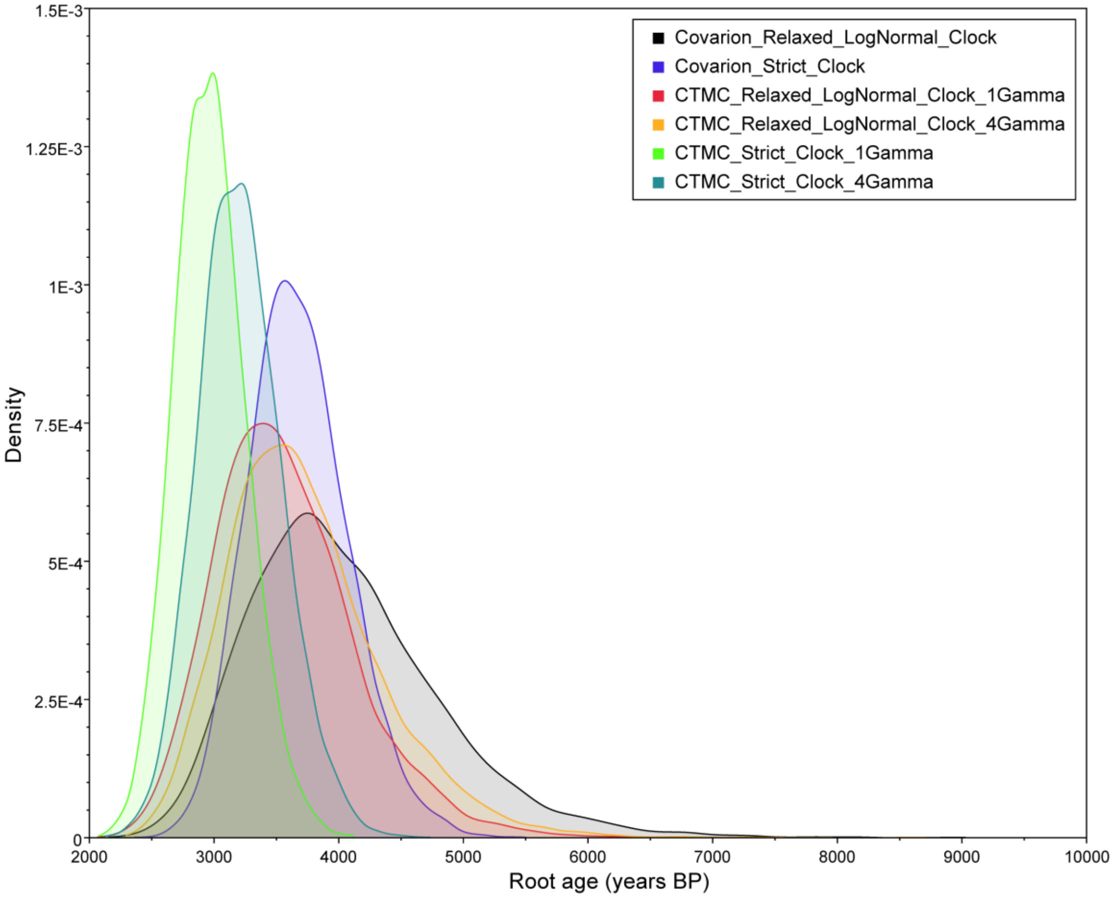

Figure S6. The distribution of the root age of the Proto-Kra-Dai language under six models. The estimated root time for Kra-Dai languages under six combinations of models. Each model was run for 50,000,000 generations, in a sampling frequency of 5,000, with a burn-in of the first 10% of samples.

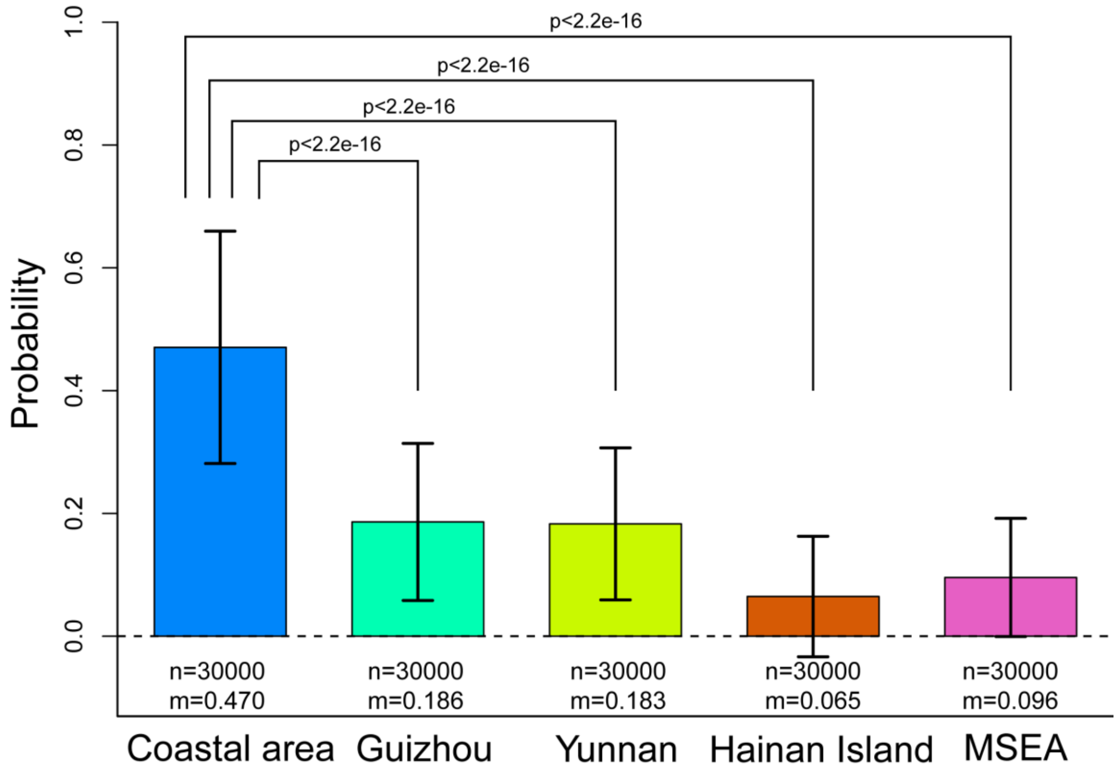

Figure S7. Probabilities of geographical distribution for the root of Kra-Dai languages. Error bars indicated standard deviation (SD). Data are presented as mean values +/− SD. Values ranged from 0 to 1. More details for the data see Supplementary Data 11. The significance tested by paired one-side Wilcoxon signed rank test was indicated by exact p value. $P < 0.05$ indicated that the probability of coastal area for the root of Kra-Dai languages is significantly higher than others.

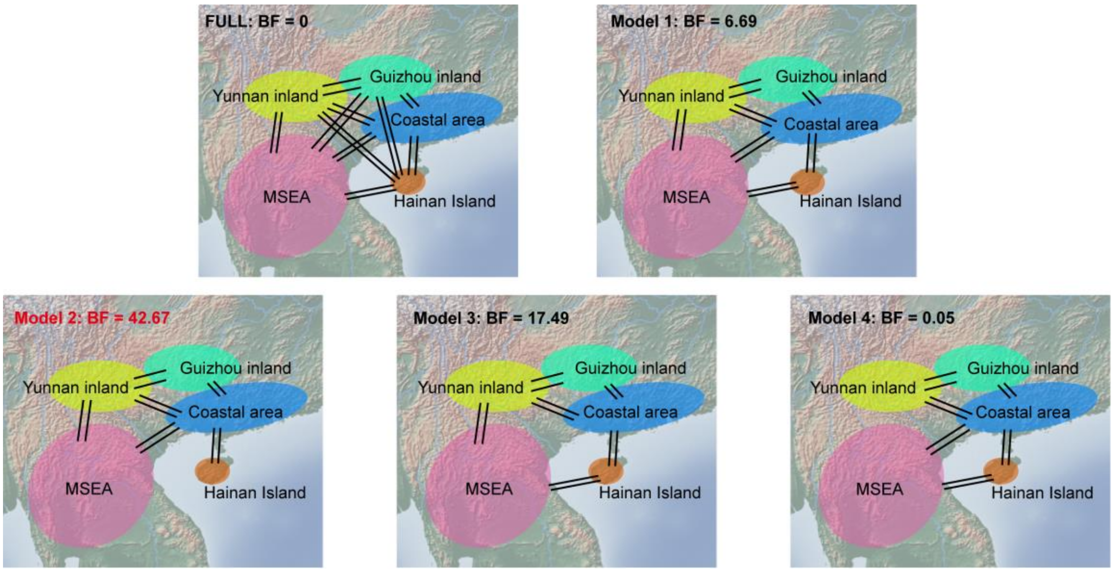

Figure S8. Models of dispersal routes tested in this study. The FULL model allows transitions between any two of the areas. Model 1 disallows transitions between geographically isolated regions. Models 2, 3, and 4 are defined with more constrains based on Model 1. Model 2 disallows the transitions between MSEA and Hainan Island. Model 3 disallows the transitions between MSEA and the coastal area. Model 4 disallows the transition between MSEA and Yunnan inland. Bayes Factor is the ratio of posterior to prior odds of each model in the RJMCMC analyses. The optimum model is Model 2 with the maximum Bayes Factor. The base map was derived from the vector map data from https://www.naturalearthdata.com.

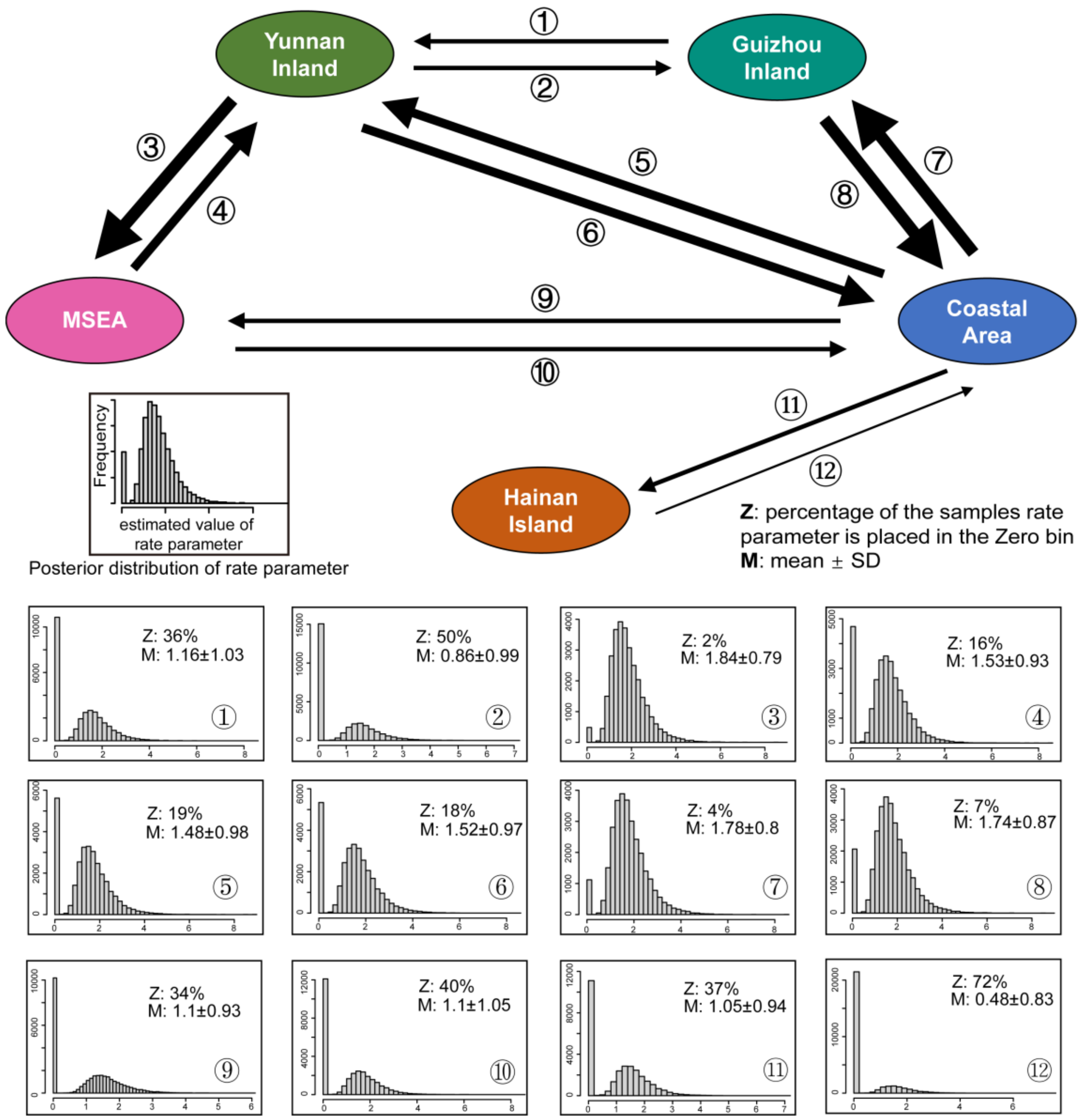

Figure S9. Estimated instantaneous rates of change between areas from the RJMCMC analysis. Only the transitional direction allowed in model 2 was labeled here. Histograms show the posterior distribution of estimated values of the rate parameters. Arrow width is divided into four groups according to Z (the percentage of samples in which each rate parameter is estimated as zero): Z ⩽ 10% is the widest; 10% < Z ⩽ 20% is the second widest; 20% < Z ⩽ 50% is the third widest; Z > 50% is the thinnest.

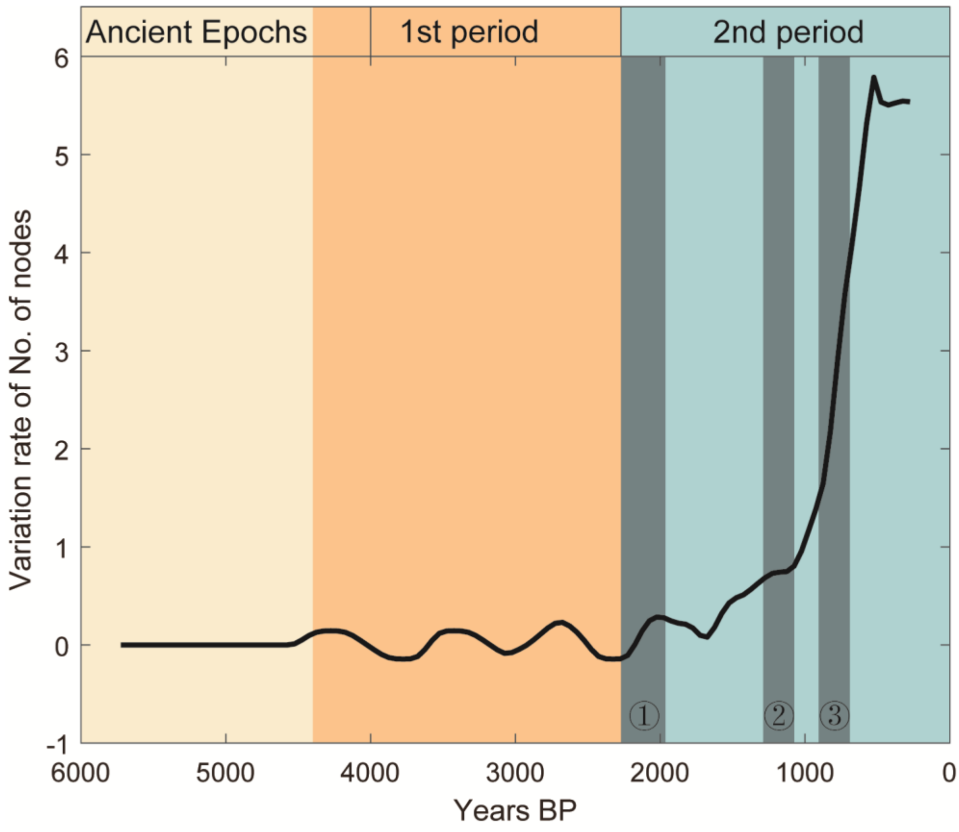

Figure S10. The variation rate of the number of nodes of Kra-Dai phylogeny. The first-order differential curve of language diversification originally in Figure 3a was shown. The three intervals marked by dark bands in the 2nd period were three important historical events that related to Kra-Dai-speaking populations, respectively.

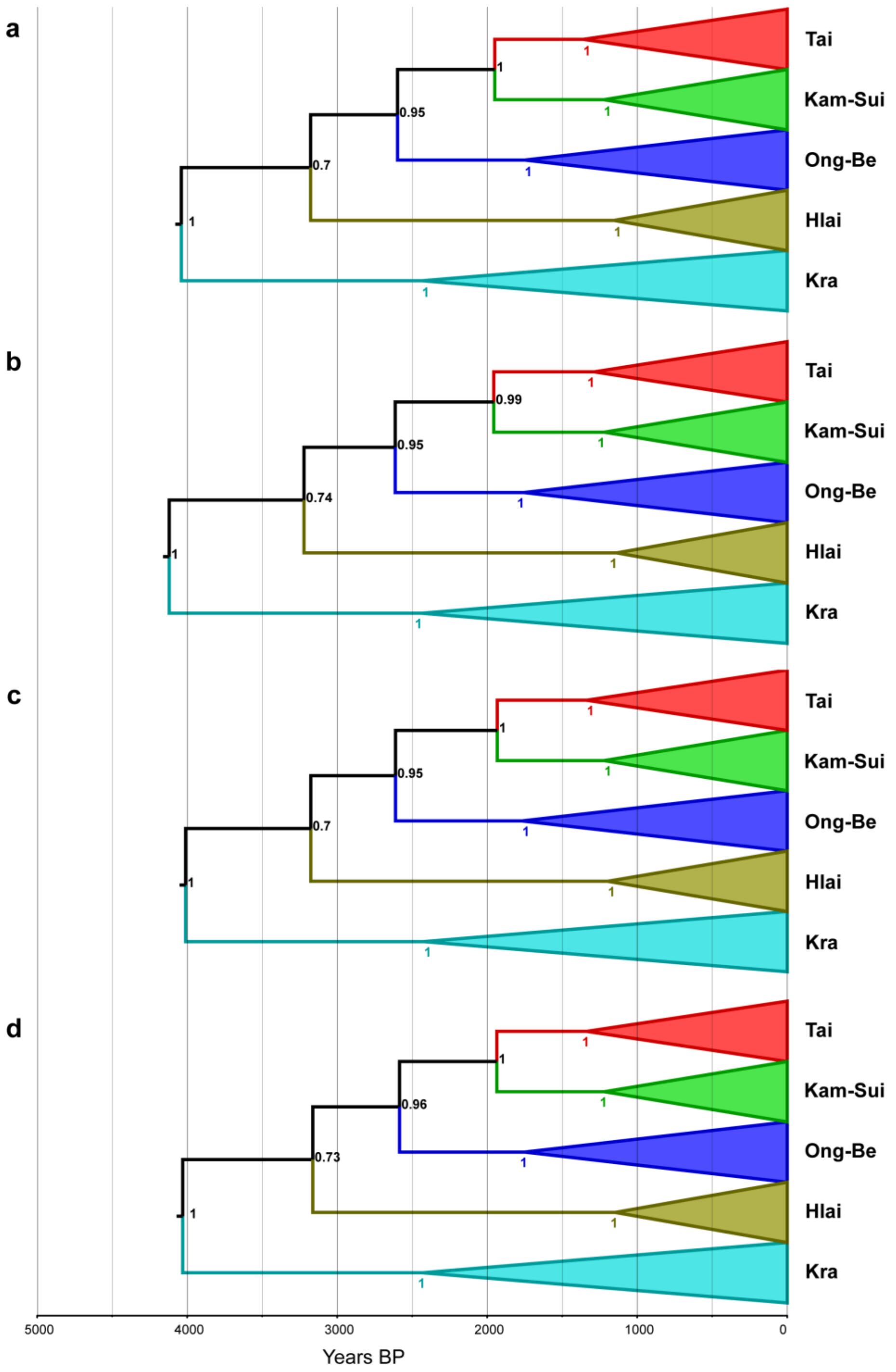

Figure S11. Comparison of linguistic relatedness of the five language branches among versions of different settings. The maximum clade credibility trees under four versions of settings were shown with the posterior values of every high-level node. Trees were reconstructed under the Covarion model and the Relaxed Lognormal clock model. (**a**) default settings (version in the manuscript); (**b**) constraining the languages of the same groups as monophyletic groups, respectively (i.e., Kra, Hlai, Ong-Be, Kam-Sui, Southwestern Tai, Central Tai, and Northern Tai); (**c**) excluding Saek from our data; (**d**) constraining the six varieties of Shan language as a monophyletic group.

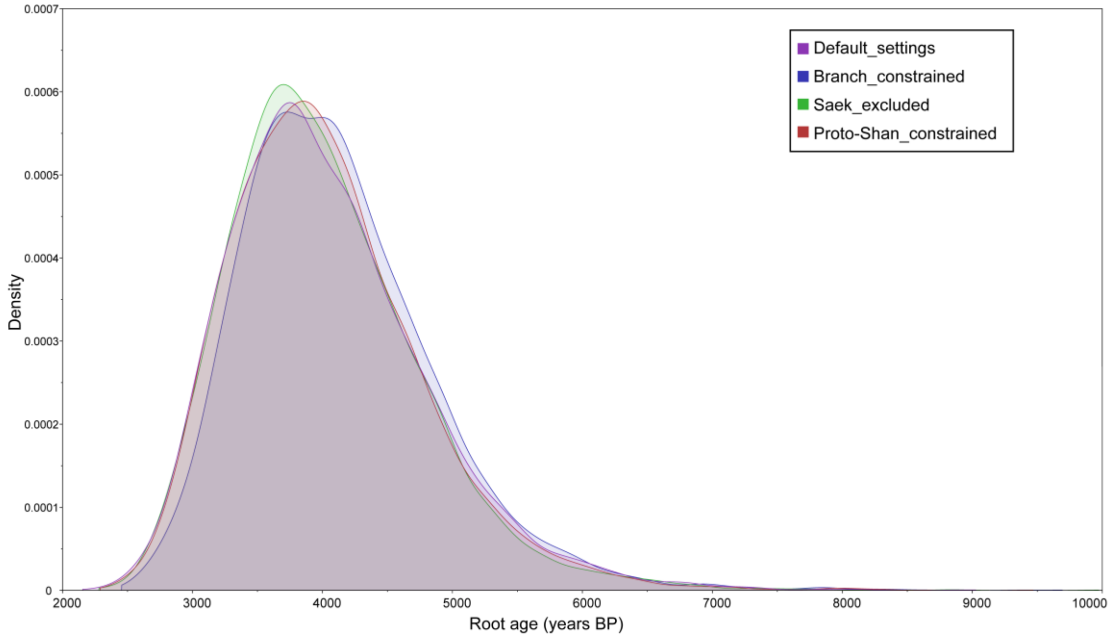

Figure S12. Comparison of the root age distributions of Kra-Dai languages among versions of different settings. Trees were reconstructed under the Covarion model and the Relaxed Lognormal clock model.

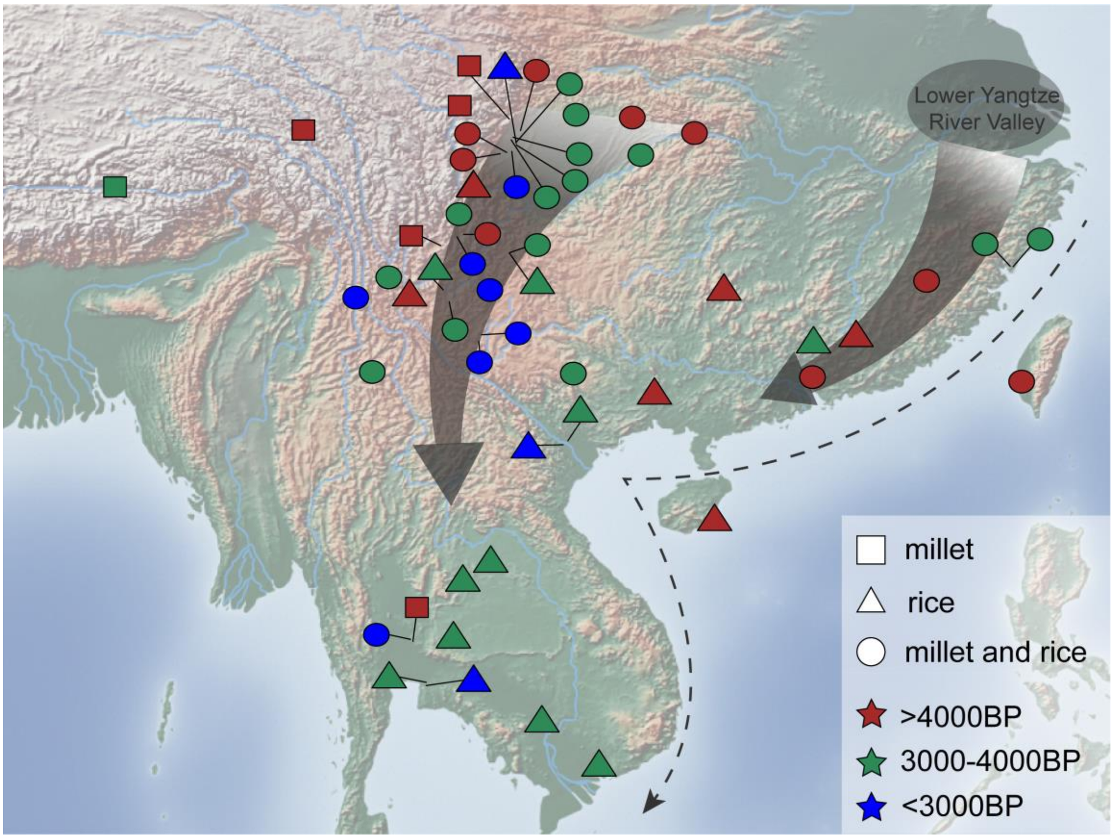

Figure S13. Agriculture spread pattern in South China and Mainland Southeast Asia. The median age of the archaeological site is used. Hundreds of sites dated to 9,000 - 3,000 years BP are illustrated as an entirety in the Lower Yangtze region, where rice was originally domesticated before 6,000 years BP and intensive agriculture continuously developed[109,110]. The two thick arrows reflected a north-to-south and east-to-west pattern for the land routes of agricultural dispersal. The dashed line indicated a possible maritime route. The base map was derived from the vector map data from https://www.naturalearthdata.com.

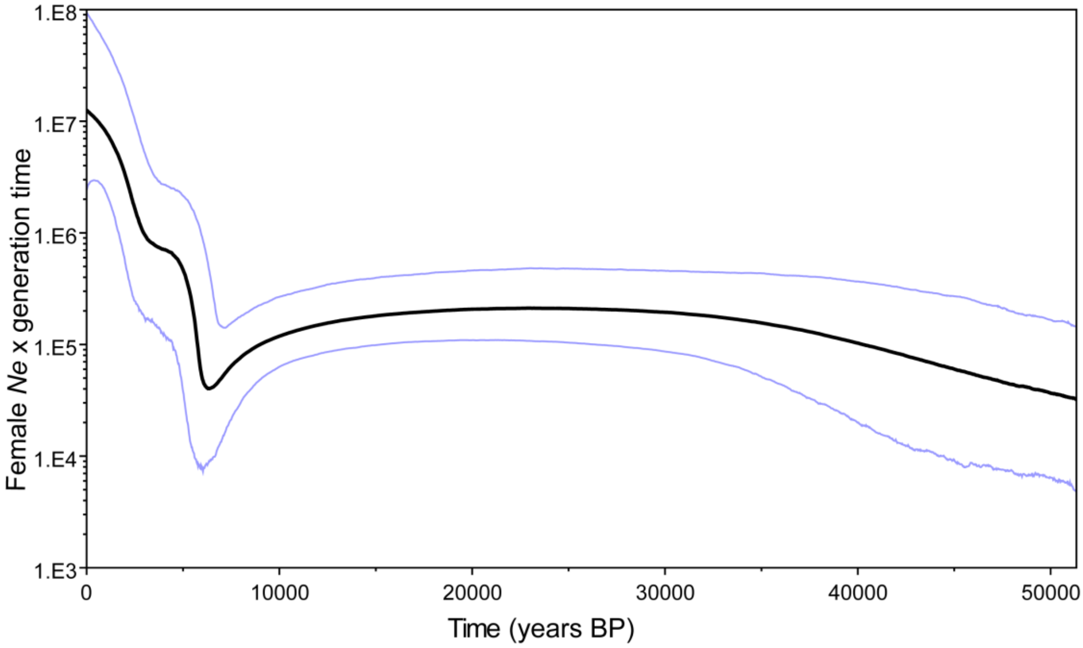

Figure S14. The effective population size of 22 representative maternal lineages of Kra-Dai-speaking populations. The black line represented the median value and the purple lines represented the upper and lower value.

References

1 Peiros, I. *Comparative Linguistics in Southeast Asia*. (Pacific Linguistics C-142, 1998).

2 Liang, M. & Zhang, J. R. *An Introduction to the Kam-Tai Languages (Chinese version)*. (China Social Sciences Press, 1996).

3 Crystal, D. *A Dictionary of Linguistics and Phonetics (6th ed.)*. 104, 418 (Blackwell Publishing, 2011).

4 Li, F. K. *A handbook of comparative Tai*. (1977).

5 Ferlus, M. REMARQUES SUR LE CONSONANTISME DU PROTO THAI-YAY (Révision du proto tai de LI Fangkuei). *23rd International Conference on Sino-Tibetan Languages and Linguistics* (1990).

6 Pittayaporn, P. *The phonology of proto-Tai*, Cornell University, (2009).

7 Pittayaporn, P. Proto-Southwestern-Tai revised: A new reconstruction. *JSEALS*, 119 (2009).

8 Ostapirat, W. The rime system of Proto-Tai. *Bulletin of Chinese Linguistics* **7**, 189-227 (2013).

9 Thurgood, G. Notes on the reconstruction of Proto-Kam-Sui. *Comparative Kadai: linguistic studies beyond Tai*, 179-218 (1988).

10 Edmondson, J. A. & Yang, Q. Word - initial Preconsonants and the History of Kam-Sui Resonant Initials and Tones. *Comparative Kadai: Linguistic studies beyond Tai*, 143-166 (1988).

11 Ferlus, M. Remarques sur le consonantisme du proto kam-sui. *Cahiers de linguistique-Asie Orientale* **25**, 235-278 (1996).

12 Zeng, X. Y. *Hanyu Shuiyu Guanxici Yanjiu (Chinese version)*. (Chongqing Publishing Group, 1996).

13 Huang, Y. *Hanyu Dongyu Guanxici Yanjiu (Chinese version)*. (Tianjin Guji Press, 2002).

14 Ostapirat, W. Alternation of tonal series and the reconstruction of Proto-Kam-Sui. *Dah-an Ho, H. Samuel Cheung, Wuyun Pan, & Fuxiang Wu(eds.), Linguistic studies in Chinese and Neighboring languages: Festschrift in Honor of Professor Pang-Hsin Ting on His 70th Birthday*, 1077-1121 (2006).

15 Long, R.-T. Research on the Tone Merger of Kam Language (Chinese version). *Guizhou Ethnic Studies* **39**, 194-199 (2018).

16 L-Thongkum, T. A Preliminary reconstruction of Proto-Lakkja (Cha Shan Yao). *The Mon-Khmer Studies Journal* **20**, 57-90 (1992).

17 Matisoff, J. A. Proto-Hlai initials and tones: a first approximation. *Comparative Kadai: linguistic studies beyond Tai*, 289-321 (1988).

18 Ostapirat, W. Proto-Hlai sound system and lexicons. *Studies on Sino-Tibetan languages: Papers in honor of Professor Hwang-cherng Gong on his seventieth birthday*, 121-175 (2004).

19 Norquest, P. K. *A phonological reconstruction of Proto-Hlai*. (The University of Arizona, 2007).

20 Chen, Y.-l. *Proto-Ong-Be*, University of Hawai'i at Manoa, (2018).

21 Ostapirat, W. *Proto-Kra*. (University of California, Berkeley, 1999).

22 Campbell, L. *Historical linguistics: An Introduction.* 4 edn, (Edinburgh University Press, 2020).

23 Trask, R. L. & Trask, R. L. *Historical linguistics*. (Oxford University Press, 1996).

24 Chang, W., Hall, D., Cathcart, C. & Garrett, A. Ancestry-constrained phylogenetic analysis supports the Indo-European steppe hypothesis. *Language*, 194-244 (2015).

25 Barido-Sottani, J. *et al.* Taming the BEAST—A community teaching material resource for BEAST 2. *Systematic biology* **67**, 170-174 (2018).

26 Haspelmath, M. Loanword typology: Steps toward a systematic crosslinguistic study of lexical

borrowability. *Empirical Approaches to Language Typology*, 43-62 (2008).
27 Zhang, J. R. Guangxi Zhongnanbu Diqu Zhuangyu zhong de Lao Jieci Yuanyu Hanyu Gu "Pinghua" Kao (Chinese version). *Studies in Language and Linguistics*, 197-219 (1982).
28 Zhang, J. R. Guangxi Pinghua zhong de Zhuangyu Jieci (Chinese version). *Studies in Language and Linguistics*, 185-189 (1987).
29 Zhang, J. R. Guangxi Pinghua dui Dangdi Zhuangdong Yuzu Yuyan de Yingxiang (Chinese version). *Minority Languages of China*, 51-56 (1988).
30 Shao, L. *A Study of Jizhao Language in Guangdong (Chinese version)*, GuangXi University for Nationalities, (2016).
31 Li, J. F. & Wu, Y. A Grammatical Sketch of the Jizhao Language Spoken in Wuchuan of Guangdong Province (Chinese version). *Minority Languages of China*, 77-96 (2017).
32 Wang, W., Fu, C. & Wei, Y. Ｒevisiting the Family of Jizhaohai Dialect (Chinese version). *Bulletin of Linguistic Studies*, 391-404+447 (2020).
33 Ding, B. X. *Chinese Dialects and Historical Strata (Chinese version)*. (Shanghai Educational Publishing House, 2007).
34 Ni, D. *An introduction to Kam-Tai languages (Chinese version)*. (China Minzu University Press, 1990).
35 Xing, G. W. *Upper Hongjin Dai Ya Language (Chinese version)*. (Language Publishing House, 1989).
36 Zhou, Y. *Daiyu Fangyan Yanjiu (Chinese version)*. (The Ethnic Publishing House, 2001).
37 Gedney, W. J. & H., T. J. *William J. Gedney's Central Tai Dialects: Glossaries, Texts, and Translations (No. 43)*. (University of Michigan Press, 1995).
38 Zhang, J. r. *Zhuangyu Fangyan Yanjiu (Chinese version)*. (Sichuan Minzu Publishing House, 1999).
39 Hudak, T. J. *William J. Gedney's comparative Tai source book*. Vol. 34 (University of Hawaii Press, 2007).
40 Li, F. K. A tentative classification of Tai dialects. *Culture in history: Essays in honor of Paul Radin*, 951-959 (1960).
41 Gray, R. D. & Atkinson, Q. D. Language-tree divergence times support the Anatolian theory of Indo-European origin. *Nature* **426**, 435-439 (2003).
42 Bouckaert, R. *et al.* Mapping the origins and expansion of the Indo-European language family. *Science* **337**, 957-960, doi:10.1126/science.1219669 (2012).
43 Tuffley, C. & Steel, M. Modeling the covarion hypothesis of nucleotide substitution. *Mathematical biosciences* **147**, 63-91 (1998).
44 Penny, D., McComish, B. J., Charleston, M. A. & Hendy, M. D. Mathematical elegance with biochemical realism: the covarion model of molecular evolution. *Journal of Molecular Evolution* **53**, 711-723 (2001).
45 Kolipakam, V. *et al.* A Bayesian phylogenetic study of the Dravidian language family. *Royal Society open science* **5**, 171504 (2018).
46 Nicholls, G. K. & Gray, R. D. Quantifying uncertainty in a stochastic model of vocabulary evolution. *Phylogenetic methods and the prehistory of languages*, 161-171 (2006).
47 Alekseyenko, A. V., Lee, C. J. & Suchard, M. A. Wagner and Dollo: a stochastic duet by composing two parsimonious solos. *Systematic biology* **57**, 772-784 (2008).
48 Pagel, M. & Meade, A. Bayesian analysis of correlated evolution of discrete characters by

reversible-jump Markov chain Monte Carlo. *The American Naturalist* **167**, 808-825 (2006).

49 Li, H. & Durbin, R. Fast and accurate short read alignment with Burrows-Wheeler transform. *Bioinformatics* **25**, 1754-1760, doi:10.1093/bioinformatics/btp324 (2009).

50 Li, H. *et al.* The Sequence Alignment/Map format and SAMtools. *Bioinformatics* **25**, 2078-2079, doi:10.1093/bioinformatics/btp352 (2009).

51 McKenna, A. *et al.* The Genome Analysis Toolkit: a MapReduce framework for analyzing next-generation DNA sequencing data. *Genome Res* **20**, 1297-1303 (2010).

52 Kong, Q.-P. *et al.* Updating the East Asian mtDNA phylogeny: a prerequisite for the identification of pathogenic mutations. *Human molecular genetics* **15**, 2076-2086 (2006).

53 Yang, X. *et al.* Mitochondrial DNA polymorphisms are associated with the longevity in the Guangxi Bama population of China. *Molecular biology reports* **39**, 9123-9131 (2012).

54 The 1000 Genomes Project Consortium. A global reference for human genetic variation. *Nature* **526**, 68-74 (2015).

55 Kutanan, W. *et al.* Complete mitochondrial genomes of Thai and Lao populations indicate an ancient origin of Austroasiatic groups and demic diffusion in the spread of Tai–Kadai languages. *Human genetics* **136**, 85-98 (2017).

56 Duong, N. T. *et al.* Complete human mtDNA genome sequences from Vietnam and the phylogeography of Mainland Southeast Asia. *Scientific reports* **8**, 1-13 (2018).

57 Kutanan, W. *et al.* New insights from Thailand into the maternal genetic history of Mainland Southeast Asia. *European Journal of Human Genetics* **26**, 898-911 (2018).

58 Edgar, R. C. MUSCLE: multiple sequence alignment with high accuracy and high throughput. *Nucleic acids research* **32**, 1792-1797 (2004).

59 Behar, D. M. *et al.* A "Copernican" reassessment of the human mitochondrial DNA tree from its root. *The American Journal of Human Genetics* **90**, 675-684 (2012).

60 Guindon, S. & Gascuel, O. A simple, fast, and accurate algorithm to estimate large phylogenies by maximum likelihood. *Systematic biology* **52**, 696-704 (2003).

61 Hosner, D., Wagner, M., Tarasov, P. E., Chen, X. & Leipe, C. Spatiotemporal distribution patterns of archaeological sites in China during the Neolithic and Bronze Age: An overview. *The Holocene* **26**, 1576-1593 (2016).

62 Gutaker, R. M. *et al.* Genomic history and ecology of the geographic spread of rice. *Nat Plants* **6**, 492-502 (2020).

63 d'Alpoim Guedes, J. & Bocinsky, R. K. Climate change stimulated agricultural innovation and exchange across Asia. *Science advances* **4**, eaar4491 (2018).

64 Walker, M. J. *et al.* Formal subdivision of the Holocene Series/Epoch: a Discussion Paper by a Working Group of INTIMATE (Integration of ice-core, marine and terrestrial records) and the Subcommission on Quaternary Stratigraphy (International Commission on Stratigraphy). *Journal of Quaternary Science* **27**, 649-659 (2012).

65 Fang, X. & Hou, G. Synthetically Reconstructed Holocene Temperature Change in China (Chinese version). *Scientia Geographica Sinica* **31**, 385-393 (2011).

66 Hou, G. & Fang, X. Characteristics of Holocene Temperature Change in China (Chinese version). *Progress in Geography* **30**, 1075-1080 (2011).

67 Ostapirat, W. in *The Peopling of East Asia: Putting together archaeology, linguistics and genetics, Kra-dai and Austronesian: notes on phonological correspondences and vocabulary distribution* 107-131 (Routledge, 2005).

68 Edmondson, J. A. & Solnit, D. B. *Comparative Kadai: the Tai branch*. (Summer Institute of Linguistics Publications in Linguistics, 1997).

69 Diller, A., Edmondson, J. & Luo, Y. *The Tai-Kadai Languages*. (Routledge, 2008).

70 Chamberlain, J. R. Kra-Dai and the proto-history of South China and Vietnam. *The Journal of the Siam Society* **104**, 27-77 (2016).

71 Ostapirat, W. Kra-Dai in Southern China. (Nankai University, 2017).

72 Li, P. J.-k. Dongdai yuzu de zujudi, kuosan ji qi shiqianwenhua (Chinese version). *Journal of East Linguistics* (2019).

73 Sidwell, P. & Jenny, M. *The Languages and Linguistics of Mainland Southeast Asia: A Comprehensive Guide*. Vol. 8 (Walter de Gruyter GmbH & Co KG, 2021).

74 Hansell, M. The relationship of Be to Tai. *Comparative Kadai: Linguistic studies beyond Tai*, 239-287 (1988).

75 Gedney, W. J. in *Michigan papers on South and Southeast Asia* Vol. 29 (Ann Arbor: Center for South and Southeast Asian Studies, 1989).

76 Haudricourt, A.-G. De la restitution des initiales dans les langues monosyllabiques: le problème du thai commun. *Bulletin de la Société de Linguistique de Paris* **52**, 307-322 (1956).

77 Chamberlain, J. R. in *Studies in Tai linguistics in honor of William J. Gedney.* 49-60 (Office of State Universities).

78 Strecker, D. in *Southeast Asian Linguistic Studies presented to André G. Haudricourt.* 479-492 (Bangkok: Mahidol University).

79 Ferlus, M. Remarques sur le consonantisme du proto thai-yay. *Circulated at the 23d ICSTLL, Arlington, Texas* (1990).

80 Edmondson, J. A. in *46th International Conference on Sino-Tibetan Languages and Linguistics (ICSTLL 46), Dartmouth College, Hanover, New Hampshire, United States.* 7-10.

81 Ostapirat, W. A Mainland Bê Language? *Journal of Chinese Linguistics* **26**, 338-344 (1998).

82 Solnit, D. B. The position of Lakkia within Kadai. *Comparative Kadai: Linguistic studies beyond Tai*, 219-238 (1988).

83 Brown, J. M. *From ancient Thai to modern dialects and other writings on historical Thai linguistics*. (White Lotus Company, 1985).

84 He, P. Miandian Fengjianwangchao Shili de Beikuo yu Danbang de Xingcheng (Chinese version). *South and Southeast Asian Studies*, 44-52 (2003).

85 Gogoi, P., Morey, S. & Pittayaporn, P. The Tai Ahom sound system as reflected by the texts recorded in the bark manuscripts. *Journal of the Southeast Asian Linguistics Society* **13**, 14-42 (2020).

86 Khanittanan, W. & Yang, G. Saek Language (Part One) (Chinese version). *Nankai Linguistics*, 154-181+187 (2003).

87 Khanittanan, W. & Yang, G. Saek Language (Part Two) (Chinese version). *Nankai Linguistics*, 171-196 (2004).

88 Zhang, M., Yan, S., Pan, W. & Jin, L. Phylogenetic evidence for Sino-Tibetan origin in northern China in the Late Neolithic. *Nature* **569**, 112-115, doi:10.1038/s41586-019-1153-z (2019).

89 Atkinson, Q. D. & Gray, R. D. Curious parallels and curious connections—phylogenetic thinking in biology and historical linguistics. *Systematic biology* **54**, 513-526 (2005).

90 Qin, S.-m. The Origin of the Zhuang-Thai Ethnic Groups (Chinese version). *Journal of Guangxi University for Nationalities (Philosophy and Social Science Edition)* **27**, 110-117 (2005).

91 Jiang, W. The relationship between culture and language. *ELT journal* **54**, 328-334 (2000).
92 Gray, R. D., Drummond, A. J. & Greenhill, S. J. Language phylogenies reveal expansion pulses and pauses in Pacific settlement. *Science* **323**, 479-483 (2009).
93 Grollemund, R. *et al.* Bantu expansion shows that habitat alters the route and pace of human dispersals. *Proceedings of the National Academy of Sciences* **112**, 13296-13301 (2015).
94 Bouckaert, R. R., Bowern, C. & Atkinson, Q. D. The origin and expansion of Pama–Nyungan languages across Australia. *Nature ecology & evolution* **2**, 741-749 (2018).
95 Sagart, L. *et al.* Dated language phylogenies shed light on the ancestry of Sino-Tibetan. *Proceedings of the National Academy of Sciences* **116**, 10317-10322 (2019).
96 Pereltsvaig, A. & Lewis, M. W. *The Indo-European Controversy: Facts and Fallacies in Historical Linguistics*. (Cambridge University Press, 2015).
97 Zhang, M., Zheng, H. X., Yan, S. & Jin, L. Reconciling the father tongue and mother tongue hypotheses in Indo-European populations. *Natl Sci Rev* **6**, 293-300 (2019).
98 Greenhill, S. J. *et al.* Evolutionary dynamics of language systems. *Proceedings of the National Academy of Sciences* **114**, E8822-E8829 (2017).
99 Thomason, S. G. & Kaufman, T. *Language contact*. (Edinburgh University Press, 2001).
100 Greenhill, S. J., Heggarty, P. & Gray, R. D. in *The handbook of historical linguistics* Vol. 2 *Bayesian Phylolinguistics* Ch. 11, 226-253 (2020).
101 Ringe, D., Warnow, T. & Taylor, A. Indo-European and computational cladistics. *Transactions of the philological society* **100**, 59-129 (2002).
102 Hung, H.-c. in *Handbook of East and Southeast Asian archaeology* 633-658 (Springer, 2017).
103 Bouckaert, R. R. & Heled, J. DensiTree 2: Seeing trees through the forest. *BioRxiv*, 012401 (2014).
104 Greenhill, S. J. & Gray, R. D. Basic vocabulary and Bayesian phylolinguistics: Issues of understanding and representation. *Diachronica* **29**, 523-537 (2012).
105 He, P. The Changes of the Frontier Region of Southwest China and the Origin of the Shans in Burma (Chinese version). *Journal of Yunnan Minzu University (Philosophy and Social Sciences Edition)*, 84-89 (2007).
106 Neureiter, N. *et al.* Detecting contact in language trees: a Bayesian phylogenetic model with horizontal transfer. *Humanities and Social Sciences Communications* **9**, 1-14 (2022).
107 Koile, E., Greenhill, S. J., Blasi, D. E., Bouckaert, R. & Gray, R. D. Phylogeographic analysis of the Bantu language expansion supports a rainforest route. *Proc Natl Acad Sci U S A* **119**, e2112853119 (2022).
108 Bellwood, P. *First Farmers: the Origins of Agricultural Societies*. (Blackwell Publishing, 2007).
109 Fuller, D. Q. *et al.* The domestication process and domestication rate in rice: spikelet bases from the Lower Yangtze. *Science* **323**, 1607-1610 (2009).
110 Long, T., Chen, H., Leipe, C., Wagner, M. & Tarasov, P. E. Modelling the chronology and dynamics of the spread of Asian rice from ca. 8000 BCE to 1000 CE. *Quaternary International* **623**, 101-109 (2022).
111 He, K., Lu, H., Zhang, J., Wang, C. & Huan, X. Prehistoric evolution of the dualistic structure mixed rice and millet farming in China. *The Holocene* **27**, 1885-1898 (2017).
112 Tang, Y., Marston, J. M. & Fang, X. Early millet cultivation, subsistence diversity, and wild plant use at Neolithic Anle, Lower Yangtze, China. *The Holocene*, doi:10.1177/09596836221109004 (2022).

113 Lambeck, K., Rouby, H., Purcell, A., Sun, Y. & Sambridge, M. Sea level and global ice volumes from the Last Glacial Maximum to the Holocene. *Proc Natl Acad Sci U S A* **111**, 15296-15303, doi:10.1073/pnas.1411762111 (2014).
114 He, G. *et al.* Inferring the population history of Tai-Kadai-speaking people and southernmost Han Chinese on Hainan Island by genome-wide array genotyping. *European Journal of Human Genetics* **28**, 1111-1123 (2020).
115 Wang, M. G. *et al.* Reconstructing the genetic admixture history of Tai-Kadai and Sinitic people: Insights from genome-wide SNP data from South China. *Journal of Systematics and Evolution* (2021).
116 Hung, H.-c. Prosperity and complexity without farming: the South China Coast, c. 5000–3000 BC. *Antiquity* **93**, 325-341 (2019).
117 Zhang, X. *et al.* A matrilineal genetic perspective of hanging coffin custom in Southern China and Northern Thailand. *Iscience* **23**, 101032 (2020).
118 Matsumura, H. *et al.* Craniometrics reveal "two layers" of prehistoric human dispersal in eastern Eurasia. *Scientific Reports* **9**, 1-12 (2019).
119 Yang, M. A. *et al.* Ancient DNA indicates human population shifts and admixture in northern and southern China. *Science* **369**, 282-288 (2020).
120 Wang, C.-C. *et al.* Genomic insights into the formation of human populations in East Asia. *Nature* **591**, 413-419 (2021).
121 Wang, T. *et al.* Human population history at the crossroads of East and Southeast Asia since 11,000 years ago. *Cell* **184**, 3829-3841.e3821, doi:10.1016/j.cell.2021.05.018 (2021).
122 Pan, Z. A Preliminary Comment on the Relationship among Kam-Tai Family, Baiyue and Austronesian Family (Chinese version). *Guangxi Ethnic Studies*, 131-140 (2005).
123 Guo, J., Deng, X. & Wang, C.-C. Perspectives from Archaeology and Paleogenomics on the Origin and Dispersal of Austronesian (Chinese version). *Taiwan Research Journal* (2022).
124 Hu, S. *Zhongguo Nanfang Minzu Fazhanshi (Chinese version)*. (The Ethnic Publishing House, 2004).
125 Yang, W. & Wang, C.-C. Dongtai Yuzu Qiyuan yu Kuosan de Kuaxueke Tansuo (Chinese version). *Chinese Social Sciences Today* (2022).
126 Jin, L., Seielstad, M. & Xiao, C. *Genetic, linguistic and archaeological perspectives on human diversity in Southeast Asia*. Vol. 8 (World Scientific, 2001).
127 Chen, H. *et al.* Tracing Bai-Yue Ancestry in Aboriginal Li People on Hainan Island. *Mol Biol Evol* **39** (2022).
128 Leipe, C., Long, T., Sergusheva, E., Wagner, M. & Tarasov, P. Discontinuous spread of millet agriculture in eastern Asia and prehistoric population dynamics. *Science Advances* **5**, eaax6225 (2019).
129 Lyu, H. Periodic climate change and human adaptation (Chinese version). *Acta Anthropologica Sinica* **41**, 731-748 (2022).
130 Renfrew, C. At the edge of knowability: towards a prehistory of languages. *Cambridge Archaeological Journal* **10**, 7-34 (2000).
131 Bellwood, P. S. & Renfrew, C. *Examining the farming/language dispersal hypothesis*. (McDonald Institute for Archaeological Research, 2002).
132 Chi, Z. & Hung, H.-c. The emergence of agriculture in southern China. *Antiquity* **84**, 11-25 (2010).

133 Stevens, C. J. & Fuller, D. Q. The spread of agriculture in Eastern Asia: Archaeological bases for hypothetical farmer/language dispersals. *Language Dynamics and Change* **7**, 152-186 (2017).

134 Higham, C. F. First farmers in mainland Southeast Asia. *Journal of Indo-Pacific Archaeology* **41**, 13-21 (2017).

135 Lipson, M. *et al.* Ancient genomes document multiple waves of migration in Southeast Asian prehistory. *Science* **361**, 92-95 (2018).

136 Gao, Y., Dong, G., Yang, X. & Chen, F. A review on the spread of prehistoric agriculture from southern China to mainland southeast Asia (Chinese version). *Science China Earth Sciences,* **50**, 723-734 (2020).

137 Deng, Z., Huang, B., Zhang, Q. & Zhang, M. First farmers in the South China coast: new evidence from the Gancaoling site of Guangdong Province. *Frontiers in Earth Science*, 333 (2022).

138 Hung, H. C. *et al.* Ancient jades map 3,000 years of prehistoric exchange in Southeast Asia. *Proc Natl Acad Sci U S A* **104**, 19745-19750 (2007).

139 Kutanan, W. *et al.* Contrasting paternal and maternal genetic histories of Thai and Lao populations. *Molecular Biology and Evolution* **36**, 1490-1506 (2019).

140 Weber, S., Lehman, H., Barela, T., Hawks, S. & Harriman, D. Rice or millets: early farming strategies in prehistoric central Thailand. *Archaeological and Anthropological Sciences* **2**, 79-88 (2010).

141 Li, H. *et al.* Prehistoric agriculture development in the Yunnan-Guizhou Plateau, southwest China: Archaeobotanical evidence. *Science China Earth Sciences* **59**, 1562-1573 (2016).

142 Stoneking, M. *et al.* Genomic perspectives on human dispersals during the Holocene. *Proc Natl Acad Sci U S A* **120**, e2209475119 (2023).

143 Ren, Z. *et al.* Genetic substructure of Guizhou Tai-Kadai-speaking people inferred from genome-wide single nucleotide polymorphisms data. *Frontiers in Ecology and Evolution* **10** (2022).

144 Bin, X. *et al.* Genomic Insight Into the Population Structure and Admixture History of Tai-Kadai-Speaking Sui People in Southwest China. *Front Genet* **12**, 735084 (2021).

145 Chen, J. *et al.* Fine-Scale Population Admixture Landscape of Tai-Kadai-Speaking Maonan in Southwest China Inferred From Genome-Wide SNP Data. *Front Genet* **13**, 815285 (2022).

146 Huang, X. *et al.* Genomic Insights Into the Demographic History of the Southern Chinese. *Frontiers in Ecology and Evolution* **10** (2022).

147 Kutanan, W. *et al.* Reconstructing the Human Genetic History of Mainland Southeast Asia: Insights from Genome-Wide Data from Thailand and Laos. *Mol Biol Evol* **38**, 3459-3477, doi:10.1093/molbev/msab124 (2021).

148 Liu, D. *et al.* Extensive Ethnolinguistic Diversity in Vietnam Reflects Multiple Sources of Genetic Diversity. *Mol Biol Evol* **37**, 2503-2519 (2020).

149 Holland, B. R., Huber, K. T., Dress, A. & Moulton, V. Delta plots: a tool for analyzing phylogenetic distance data. *Mol Biol Evol* **19**, 2051-2059 (2002).

150 Gray, R. D., Bryant, D. & Greenhill, S. J. On the shape and fabric of human history. *Philos Trans R Soc Lond B Biol Sci* **365**, 3923-3933, doi:10.1098/rstb.2010.0162 (2010).

151 Snel, B., Bork, P. & Huynen, M. A. Genome phylogeny based on gene content. *Nat Genet* **21**, 108-110 (1999).

152 Holman, E. W., Walker, R., Rama, T. & Wichmann, S. Correlates of Reticulation in Linguistic Phylogenies. *Language Dynamics and Change* **1**, 205-240 (2011).
