# Supplementary material for: Phylogenetic evidence reveals early Kra-Dai divergence and dispersal in the late Holocene
Source: Nat Commun. 2023 Oct 30;14:6924. doi: 10.1038/s41467-023-42761-x (PMC10616200; doi:10.1038/s41467-023-42761-x)
Supplement: Supplementary file 3 — Description of Additional Supplementary Files [file 41467_2023_42761_MOESM3_ESM.pdf]

Description of Additional Supplementary Files

Supplementary Data 1
Description: The lexical cognate database of Kra-Dai languages. Sheet 1 "Lexical items" shows the language entries and lexical items of 100 Kra-Dai languages. Sheet 2 "Binary coded sets" shows the binary coded form based on Sheet 1. Sheet 3 "Note" shows the reason for deleting specific lexical items and descriptions for the data.

Supplementary Data 2
Description: Node constraints with known historical information used to calibrate the divergence time calculations in BEAST.

Supplementary Data 3
Description: Model comparison among different combinations of models in BEAST. Sheet 1 "HME" shows the results compared by Harmonic Mean Estimator. Sheet 2 "Path Sampling" shows the results compared by the path sampling method.

Supplementary Data 4
Description: Possibilities of ancestral area.

Supplementary Data 5
Description: Comparison of time depth and root probability among versions of different settings.

Supplementary Data 6
Description: Delta scores and Q-residual scores.

Supplementary Data 7
Description: Information on archaeological sites in South China and MSEA.

Supplementary Data 8
Description: Resources of linguistic data.

Supplementary Data 9
Description: BEAST xml files, nexus files, MCC tree file, and the log file of the best-fitting model.

Supplementary Data 10
Description: Statistical significance among models of dispersal routes in the posterior samples of 3 independent runs of the RJMCMC.

Supplementary Data 11
Description: The control file, input file, and geographical distribution file used in the *BayesTraits* program. Three log files of MCMC runs were also included.

Supplementary Data 12
Description: The details of Kra-Dai population samples included in this study, including references, haplogroups, accession codes, etc.

Supplementary Data 13

Description: The interdisciplinary data and code used to plot Figure 3. The time ranges and geographic locations of the archaeological sites were from the study of Hosner *et al.* (URL: https://doi.pangaea.de/10.1594/PANGAEA.860072). The palaeoecological data were from the study of Gutaker *et al.* (URL: https://doi.org/10.1038/s41477-020-0659-6). The paleoclimatic data were from the study of Fang and Hou (DOI: 10.13249/j.cnki.sgs.2011.04.013).
